# The FGF13‐Caveolin‐1 Axis: A Key Player in the Pathogenesis of Doxorubicin‐ and D‐Galactose‐Induced Premature Cardiac Aging

**DOI:** 10.1002/advs.202501055

**Published:** 2025-04-04

**Authors:** Enzhao Shen, Yuecheng Wu, Weijian Ye, Sihang Li, Junjie Zhu, Meifan Jiang, Zhicheng Hu, Gaoyong Cao, Xiaojing Yi, Fan Li, Zhouhao Tang, Xiaokun Li, Kwang Youl Lee, Litai Jin, Xu Wang, Weitao Cong

**Affiliations:** ^1^ School of Pharmaceutical Science Wenzhou Medical University Wenzhou 325035 P. R. China; ^2^ College of Pharmacy Research Institute of Pharmaceutical Sciences Chonnam National University Gwangju 61186 Republic of Korea; ^3^ Department of Pharmacy The Second Affiliated Hospital and Yuying Children's Hospital of Wenzhou Medical University Wenzhou 325027 P. R China; ^4^ Oujiang Laboratory (Zhejiang Lab for Regenerative Medicine Vision and Brain Health) School of Pharmaceutical Science Wenzhou Medical University Wenzhou P. R. China; ^5^ Ningbo Key Laboratory of Skin Science Ningbo College of Health Sciences Ningbo 315000 P. R. China

**Keywords:** cardiac premature aging, cardiomyocyte senescence, caveolin‐1, fibroblast growth factor 13, p53 signaling

## Abstract

Delaying senescence of cardiomyocytes has garnered widespread attention as a potential target for preventing cardiovascular diseases (CVDs). FGF13 (Fibroblast growth factor 13) has been implicated in various pathophysiological processes. However, its role in premature myocardial aging and cardiomyocyte senescence remains unknown. Adeno‐associated virus 9 (AAV9) vectors expressing FGF13 and cardiac‐specific Fgf13 knockout (Fgf13KO) mice are utilized to reveal that FGF13 overexpression and deficiency exacerbated and alleviated Doxorubicin/D‐galactose‐induced myocardial aging characteristics and functional impairment, respectively. Transcriptomics are employed to identify an association between FGF13 and Caveolin‐1 (Cav1). Mechanistic studies indicated that FGF13 regulated the Cav1 promoter activity and expression through the p38/MAPK pathway and nuclear translocation of p65, as well as the binding level of PTRF to Cav1 to mediate cardiomyocyte senescence. Furthermore, Cav1 overexpression in murine hearts reversed the alleviatory effects of FGF13 deficiency on the Doxorubicin/D‐galactose‐induced myocardial aging phenotype and dysfunction. This study has demonstrated that FGF13 regulated the Cav1‐p53‐p21 axis to augment cardiomyocyte senescence and thereby exacerbated cardiac premature aging and suggests that FGF13 knockdown may be a promising approach to combat CVDs in response to aging and chemotoxicity.

## Graphical abstract

The FGF13 level is significantly downregulated in premature aging murine hearts subject to D‐galactose and Doxorubicin. FGF13 overexpression and deficiency exacerbated and alleviated Doxorubicin/D‐galactose‐induced myocardial aging characteristics and functional impairment, respectively. The overexpression of Cav1 in murine hearts reversed the inhibitory effects of FGF13 deficiency on the Doxorubicin/D‐galactose‐induced aging characteristics and functional impairment. Mechanistically, FGF13 regulated Cav1 promoter activity and expression through the p38/MAPK pathway and nuclear translocation of p65, and upregulated the binding levels of PTRF with Cav1 to mediate cardiomyocyte senescence.

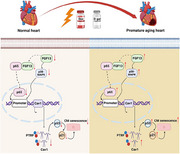

## 1 Introduction

Senescence is a major aging process that contributes to the development of CVDs.^[^ ^1^, ^2^ ^]^ Apart from replicative senescence, the senescent state can also be induced by stressors, so‐called premature senescence.^[^ ^3^ ^]^ There is increasing evidence that the gradual accumulation of senescent cardiomyocytes is causally involved in the decline of cardiovascular system function.^[^ ^4^, ^5^, ^6^ ^]^ Recent reports showed that targeting cardiomyocyte senescence can alleviate adverse remodeling after ischemia‐reperfusion and facilitate recovery.^[^ ^4^ ^]^ circHIPK3 prevents cardiac aging by recruiting a ubiquitin ligase to degrade HuR to alleviate cardiomyocyte senescence.^[^ ^5^ ^]^ Moreover, it elucidated the key role of the SIRT2‐STAT3‐CDKN2B pathway in primate cardiomyocyte senescence, revealing an acetylation switch that can reverse cardiac aging.^[^ ^6^ ^]^ Therefore, understanding the mechanisms by which cardiomyocyte senescence induces CVDs is crucial for the development of relevant therapeutic strategies.

The increasing global elderly population has led to a rise in CVDs, creating significant socio‐economic challenges.^[^ ^7^, ^8^ ^]^ However, natural aging models are limited by long timelines, poor outcomes, and high mortality,^[^ ^9^, ^10^ ^]^ prompting the use of artificial models like D‐galactose, which disrupts cardiac glucose metabolism, causing cellular senescence and premature aging.^[^ ^11^, ^12^ ^]^ Additionally, cardiotoxicity from Doxorubicin, a widely used chemotherapy drug, also induces premature aging and adverse effects in healthy tissues, including the myocardium, limiting its therapeutic potential.^[^ ^13^ ^]^ Recent advances in cardiovascular research have significantly expanded our understanding of Doxorubicin/D‐galactose‐induced premature senescence in cardiomyocytes,^[^ ^14^, ^15^ ^]^ particularly in elucidating its molecular mechanisms, associated signaling pathway alterations, and potential therapeutic interventions.

Caveolae are specialized microstructures that exist in the form of sphingolipid and cholesterol‐rich plasma membrane invaginations.^[^ ^16^ ^]^ Cav (Caveolin) is the complete protein component of Caveolae and exists as three subtypes, namely Cav1, Cav2, and Cav3.^[^ ^17^ ^]^ Among them, Cav1 is regarded as the gatekeeper of cellular senescence and is widely used to detect senescent phenotypes.^[^ ^18^ ^]^ Studies reported that Cav1 overexpression regulated tissue aging by inducing tissue‐specific dysfunction and age‐related diseases.^[^ ^19^, ^20^ ^]^ In senescent cells, the expression of Cav1 is regulated by p38‐MAPK,^[^ ^21^ ^]^Sp1,^[^ ^22^ ^]^ and NF‐κB.^[^ ^23^ ^]^ Cav1 can modulate various signaling pathways in senescent cells, including the p53/p21Waf‐1/Cip1 pathway,^[^ ^24^, ^25^ ^]^ epidermal growth factor receptor,^[^ ^26^ ^]^ and small Rho GTPase pathways.^[^ ^27^ ^]^

FGF13, a member of the non‐secretory fibroblast growth factor subfamily,^[^ ^28^ ^]^ is reportedly involved in many pathophysiological processes, including cardiac dysfunction,^[^ ^29^, ^30^ ^]^ diabetic nephropathy,^[^ ^31^ ^]^ and cancer.^[^ ^32^ ^]^ Studies showed that FGF13 was an effective regulator of NF‐κB activity in cardiomyocytes under basal and stress conditions.^[^ ^29^ ^]^ In FGF13‐deficient adult mice, Cav3 levels in cardiomyocytes were markedly elevated, protecting against chronic stress‐induced pathology.^[^ ^30^ ^]^ The FGF13 deficiency exhibited delayed neuronal migration and impaired learning and memory functions.^[^ ^33^ ^]^ FGF13 overexpression inhibited myoblast proliferation by upregulating *p27* mRNA level and downregulating Cyclin E protein expression.^[^ ^34^ ^]^ However, it remains unclear whether FGF13 is associated with cardiomyocyte senescence and premature cardiac aging.

Our research showed that the murine myocardium exhibited premature aging characteristics induced by Doxorubicin and D‐galactose, accompanied by significant downregulation of the FGF13 level. FGF13 deficiency alleviated myocardial aging characteristics and cardiac functional impairment, while FGF13 overexpression had the opposite effects. In vitro, FGF13 silencing and overexpression markedly alleviated and exacerbated Doxorubicin/D‐galactose‐induced cardiomyocyte senescence, respectively. Mechanistically, FGF13 regulated Cav1 promoter activity and expression through the p38/MAPK pathway and nuclear translocation of p65. Additionally, it also mediated p53‐p21 pathway by upregulating the binding levels of PTRF with Cav1. In summary, we demonstrated that FGF13 exacerbated cardiac premature aging through the Cav1‐p53‐p21 axis.

## 2 Results

### 2.1 The Abundance of FGF13 is Downregulated in Premature Cardiac Aging Models

**Figure 1:**
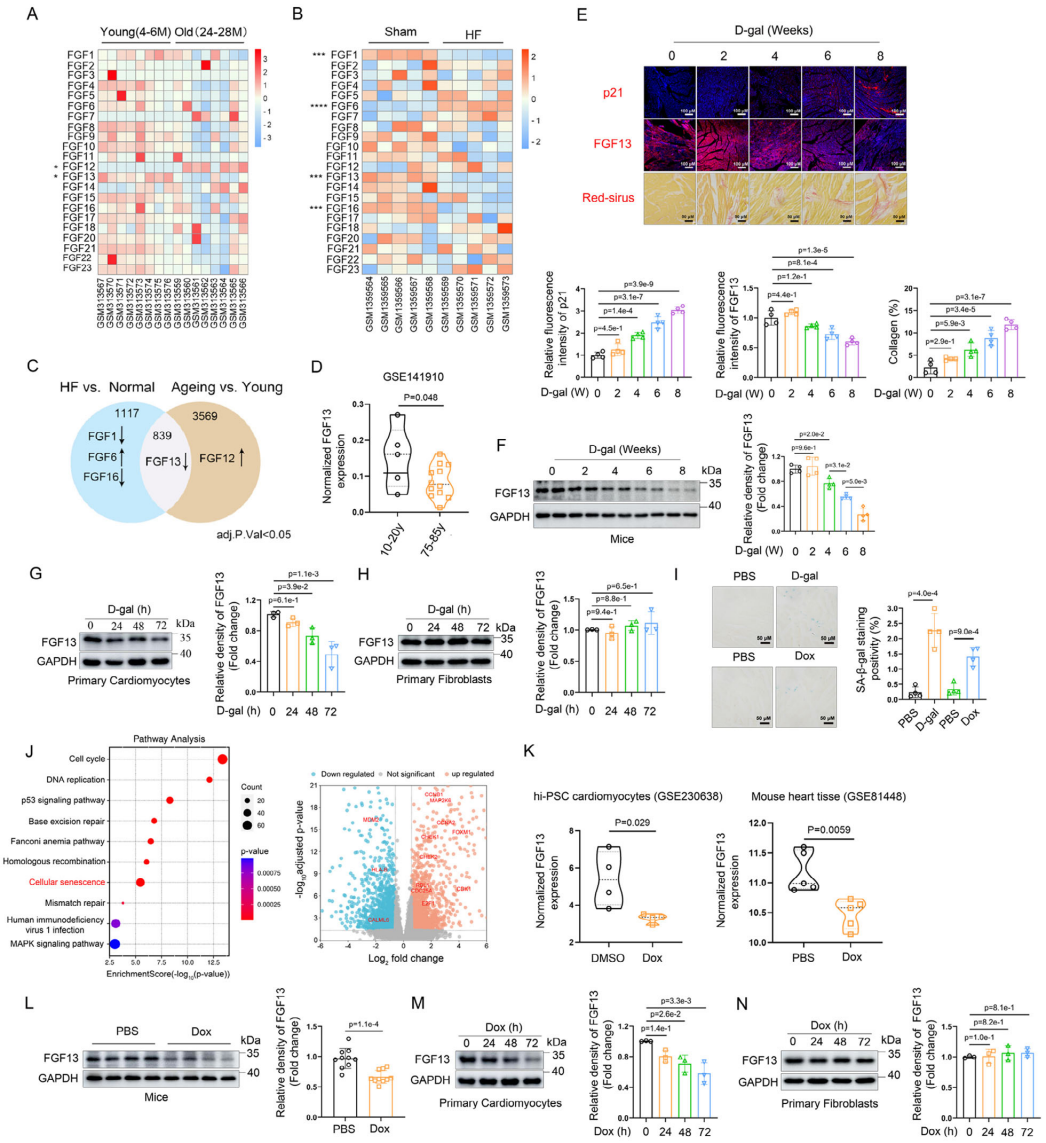
The abundance of FGF13 is downregulated in premature cardiac aging models. A–C) The results were from the analysis of the GSE12480 dataset (A) and GSE56348 dataset (B). A) The corresponding *FGFs* of the 2 genes correlated with the myocardial aging were visualized by the heatmap in the hearts from 8 young (4‐6 month) and 8 old (25–28 month) mice samples. B) The corresponding *FGFs* of the 4 genes correlated with heart failure were visualized by the heatmap in the hearts from 5 mice with heart failure (HF) and 5 sham‐operated control samples (Sham‐HF). The darker shade of red or blue represents the higher correlation level. C) Venn diagram visualizing the myocardial aging and failure‐related differentially expressed genes between two datasets. D) The relative mRNA expression level of *FGF13* in 5 human young hearts and 12 human aging hearts (from GSE141910). E,F) 8‐Week mice were administered with D‐galactose (200 mg kg^−1^ per day for 0, 2, 4, 6, 8 weeks). Myocardial tissues were collected for indicated analyses. E) Representative images of hearts stained with p21, FGF13, or Sirius red for the same groups (n = 4 per group) and the quantitative analysis of p21‐positive areas (n = 4 per group), FGF13‐positive areas (n = 4 per group), and interstitial fibrosis (Sirius red, n = 4 per group). F) Representative western blotting for FGF13 in heart tissue and the quantitative analysis (n = 4 per group). G,H) Representative western blotting for FGF13 in G) primary cardiomyocytes and H) fibroblasts after D‐galactose (20 g L^−1^) stimulation for different times (n = 3 per group) and the quantitative analysis (n = 3 per group). I) Representative image of SA‐*β‐gal* in the heart tissue, treated with D‐galactose (200 mg kg^−1^ per day for 8 weeks) and PBS (the upper part), treated with Doxorubicin (2.5 mg/kg/time × 2 per week for 4 consecutive weeks) and PBS (the bottom part), and the quantitative analysis of the percentage of SA‐*β‐gal*‐positive areas (n = 4 per group). J,K) The results were from the analysis of the GSE230638 dataset. Comparative gene expression profiling analysis of RNA‐seq data for hiPSC‐cardiomyocyte cells treated with Doxorubicin versus DMSO. J) The KEGG pathways. The x‐axis is the rich factor. The y‐axis refers to pathway terms. Volcano plot of differentially expressed genes. Red dots represent upregulated genes, blue dots represent downregulated genes, and gray dots represent genes that were not differentially expressed. We use log2 of the fold change as the source of data for the X axis and‐log10 of the P as the source of data for the y axis. Fold change >1.5× and adjusted *p*‐value <0.05 indicate statistically significant differences. The differentially expressed genes of Doxorubicin‐induced cardiomyocyte senescence pathway have been labeled. An online platform (https://www.bioinformatics.com.cn) was utilized. K) The relative mRNA expression level of *FGF13* was analyzed (from GSE230638) (n = 5 per group) and the relative mRNA expression level of *Fgf13* in 5 Doxorubicin or PBS treated murine hearts (from GSE81448). L) 8‐Week mice were administered with Doxorubicin (2.5 mg/kg/time × 2 per week for 4 consecutive weeks) and PBS. Then the myocardial tissues were collected. Representative western blotting for FGF13 in heart tissue and the quantitative analysis (n = 10 per group). M,N) Representative western blotting for FGF13 in M) cardiomyocytes and N) fibroblasts after Doxorubicin (0.1 µm) stimulation for different times and the quantitative analysis (n = 3 per group). The protein level was standardized by GAPDH. Data are means ± SEM.  The P value was determined using two‐tailed unpaired Student's t test or ANOVA with Tukey's multiple comparisons test.

To examine the correlation between expression of *FGFs* and heart ageing and heart failure, the GSE12480 and GSE56348 datasets were analyzed. The hierarchical cluster heatmap and Venn diagram indicated that 2 of 22 *FGF* genes (*FGF12* and *FGF13*) were associated with myocardial aging (**Figure** **1**A,C) and 4 of 22 *FGF* genes (*FGF1*, *FGF6*, *FGF13*, and *FGF16*) were related to myocardial failure (Figure 1B,C). Interestingly, we found that *FGF13* was markedly downregulated in both aged and failing hearts compared to control groups (Figure 1C). Consistently, it was revealed that the mRNA level of *FGF13* in aging human hearts was decreased compared to young human hearts (from GSE141910) (Figure 1D). These findings suggest that the role of FGF13 in cardiac aging and aging‐related CVDs could be further explored. Thereby, a premature cardiac aging model was induced by intraperitoneal injection of D‐galactose.^[^ ^11^, ^12^ ^]^ Immunofluorescence techniques showed that compared to the control group, the expression level of FGF13 showed a downward trend in the heart tissue during the induction process by D‐galactose (Figure 1E). Consistently, western blot techniques showed a significant downregulation of FGF13 protein levels during the D‐galactose‐induced myocardial aging process (Figure 1F). Additionally, immunofluorescence and Sirius Red staining revealed that with the D‐galactose‐induced aging model progressed, the levels of the senescence marker p21 increased, and the degree of cardiac fibrosis worsened over time (Figure 1E). SA‐*β‐gal* staining technology also detected a significant increase in senescent levels in the D‐galactose‐induced murine hearts (Figure 1I).

Subsequently, we examined which type of cardiac cells the FGF13 content is localized to during premature myocardial aging. Immunofluorescence revealed that in the murine premature aging heart, FGF13 was downregulated in cardiomyocytes, with no significant difference in fibroblasts and endothelial cells (Figure S1, Supporting Information). Then, immunoblotting analysis consistently found that the level of FGF13 was decreased in the D‐galactose‐induced primary cardiomyocytes (Figure 1G), while no changes were observed in primary fibroblasts (Figure 1H).

In recent years, robust evidence recognizes cellular senescence as an emerging key pathophysiological mechanism underlying anthracycline‐induced myocardial dysfunction.^[^ ^35^ ^]^ Next, the GSE230638 dataset was analyzed from Doxorubicin‐treated hiPSC‐cardiomyocyte cells. Kyoto Encyclopedia of Genes and Genomes (KEGG) pathway analysis demonstrated that the cellular senescence pathway was upregulated after Doxorubicin treatment and differentially expressed senescent genes were revealed by Volcano plot (Figure 1J), accompanied by a downregulated level of *FGF13* mRNA level. Moreover, the *Fgf13* mRNA level is consistently downregulated in Doxorubicin‐related hearts compared to the control group (from GSE81448) (Figure 1K).

To investigate the relationship between FGF13 and Doxorubicin‐induced premature myocardial aging, a chronic Doxorubicin‐induced cardiomyopathy model was utilized. SA‐*β‐gal* staining technology detected an increase in senescent levels in the murine hearts exposed to Doxorubicin compared to the control group (Figure 1I). Western blot techniques showed a downregulation of FGF13 protein levels in the Doxorubicin‐induced premature myocardial aging (Figure 1L). Immunofluorescence revealed that the content of FGF13 was decreased in cardiomyocytes, with no significant difference in fibroblasts and endothelial cells (Figure S2, Supporting Information). Consistently, immunoblotting found that the levels of FGF13 were downregulated in the Doxorubicin‐induced cardiomyocytes (Figure 1M), while no significant changes were observed in fibroblasts (Figure 1N). Surprisingly, similar findings were obtained in naturally aged mice. The comparative analysis between young mice (3‐4 months old) and aging mice (24–26 months old) revealed that the aging group exhibited sparse and coarse fur, brown spots on the tail, and a significant increase in heart weight. Immunofluorescence analysis further revealed that, compared to the young group, aging cardiac tissues showed a substantial upregulation of the senescence biomarker p21 and a concurrent significant downregulation of FGF13 expression in cardiomyocytes (Figure S3, Supporting Information). Taken together, these findings demonstrate that the abundance of FGF13 is downregulated in premature cardiac aging models.

### 2.2 Cardiac‐Specific Knockout of Fgf13 in Cardiomyocytes Alleviates D‐Galactose‐Induced Cardiomyocyte Senescence and Cardiac Injury

**Figure 2:**
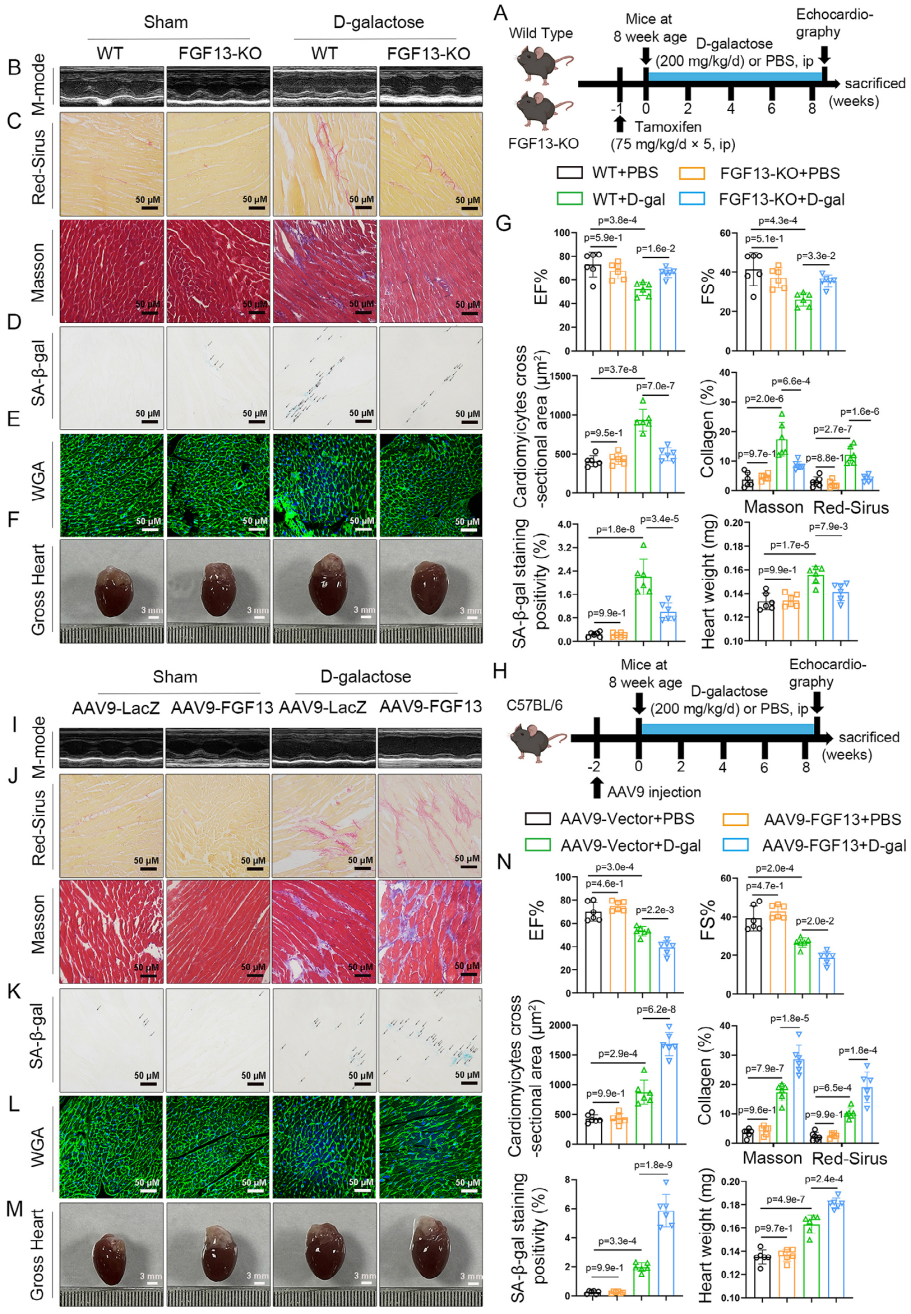
Cardiac‐specific knockout of Fgf13 in cardiomyocytes alleviates D‐galactose‐induced cardiomyocyte senescence and cardiac injury. A–G) For 7 weeks old wild‐type (WT, *Fgf13^f/Y^*) mice and *Fgf13KO* (*Fgf13^f/Y^* crossed with αMHC‐MerCreMer) mice, tamoxifen was administered at the dose of 75 mg/kg/day for 5 consecutive days. One week after the injection, mice were subjected to Sham or D‐galactose treatment. H–N) FGF13 overexpression vector (AAV9‐cTnT‐FGF13) and control vector (AAV9‐LacZ) were injected intravenously into tail veins of 6 weeks old male C57BL/ 6J mice, respectively, 2 weeks after the injection, these mice were subjected to Sham or D‐galactose treatment. A,H) The experimental flowchart. B,I) Representative M‐mode echocardiographic images from each group in mice and G,N) representative echocardiographic data for LVEF and LVFS are shown (n = 6 per group). C,J) Sirius Red staining (the upper part) (scale bar, 50 µm) and Masson staining (the lower part) (scale bar, 50 µm) and G,N) quantification (n = 6 per group). D,K) Representative image of SA‐*β‐gal* in the heart tissue (Arrows represent positive marks) (scale bar, 50 µm) and G,N) quantification (n = 6 per group). E,L) WGA (wheat germ agglutinin; scale bar, 50 µm) (n = 6 per group) and G,N) quantification (n = 6 per group). F,M) Representative whole heart images (scale bar, 3 mm) and G,N) representative data of HW in the indicated groups (n = 6 per group). Data are means ± SEM.  The P value was determined using ANOVA with Tukey's multiple comparisons test.

The myocardial‐specific *Fgf13* knockout (*Fgf13KO*) mice (Figure S4, Supporting Information) were utilized to study the role of FGF13 in D‐galactose‐induced premature cardiac aging. First, echocardiography results showed that upon treatment with D‐galactose (**Figure** **2**A), myocardial function exhibited abnormalities, characterized by left ventricular hypertrophy, and decreased ejection fraction and fractional shortening, while myocardial FGF13 deficiency mitigated these outcomes (Figure 2B,G; Table S4, Supporting Information). In addition, compared with the D‐galactose group, myocardial FGF13 deficiency reduced heart weight (HW) and the heart weight to body weight ratio (HW/BW) (Figure 2F,G; Table S4, Supporting Information). Sirius Red staining and Masson's trichrome staining revealed that the cardiac interstitial and perivascular areas exhibited an increased degree of fibrosis upon D‐galactose treatment, but myocardial FGF13 deficiency alleviated this effect (Figurea 2C,G; S6A,D, Supporting Information). Moreover, the SA‐*β‐gal* staining showed that myocardial FGF13 deficiency reduced D‐galactose‐induced premature myocardial senescent level, as indicated by a decrease in positive staining (Figure 2D,G). Wheat germ agglutinin (WGA) staining revealed that compared with the D‐galactose‐treated group, myocardial FGF13 deficiency resulted in a smaller surface area size of cardiomyocytes (Figure 2E,G). Consistent with the pathological examination and echocardiography results, western blotting demonstrated that myocardial FGF13 deficiency reduced the protein levels of p53 and p21, which are aging markers, upon D‐galactose treatment (Figure S6B,E,F, Supporting Information). In addition, immunofluorescence revealed that myocardial FGF13 deficiency showed a significant downregulation of p21 in cardiomyocytes, compared with the D‐galactose‐treated group (Figure S6C, Supporting Information). Therefore, these results suggest that myocardial FGF13 deficiency alleviates D‐galactose‐induced myocardial aging and functional impairment.

To further explore the role of FGF13 in D‐galactose‐induced premature myocardial aging, we administered an adeno‐associated virus overexpressing FGF13 (AAV9‐FGF13) via tail vein injection in mice (Figure 2H; Figure S7A, Supporting Information). Expectedly, myocardial‐specific FGF13 overexpression could further exacerbate D‐galactose‐induced impairment of myocardial function and structure (Figure 2I,N; Table S5, Supporting Information). Compared to the D‐galactose group, myocardial specific FGF13 overexpression further elevated the level of HW and HW/BW (Figure 2M,N; Table S5, Supporting Information), the degree of fibrosis (Figure 2J,N; Figure S8A–D, Supporting Information), SA‐*β‐gal* activity (Figure 2K,N), and cardiomyocyte surface area (Figure 2L,N). In addition, it also showed that FGF13 overexpression further increased the p53 and p21 protein levels (Figure S8B,E,F, Supporting Information), *p21* mRNA level (Figure S18A, Supporting Information), and the p21 content of cardiomyocytes (Figure S8C, Supporting Information) upon D‐galactose treatment. It was concluded that myocardial FGF13 deficiency alleviated D‐galactose‐induced myocardial senescent level and functional impairment.

### 2.3 Cardiac‐Specific Knockout of Fgf13 in Cardiomyocytes Alleviates Doxorubicin‐Induced Cardiomyocyte Senescence and Cardiac Injury

**Figure 3:**
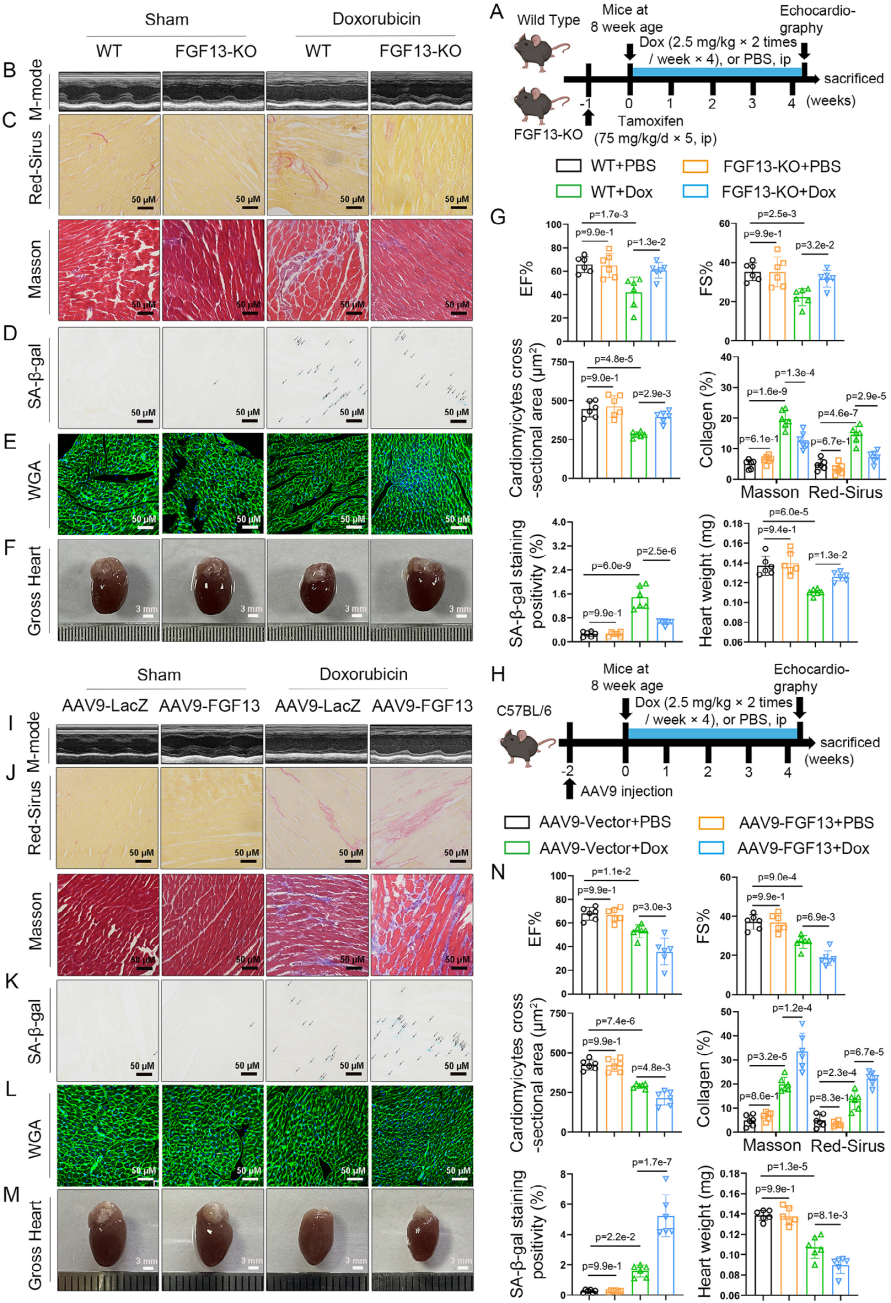
Cardiac‐specific knockout of Fgf13 in cardiomyocytes alleviates Doxorubicin‐induced cardiomyocyte senescence and cardiac injury. A–G) For 7 weeks old wild‐type (WT, *Fgf13^f/Y^*) mice and *Fgf13KO* (*Fgf13^f/Y^* crossed with αMHC‐MerCreMer) mice, tamoxifen was administered at the dose of 75 mg/kg/day for 5 consecutive days. One week after the injection, these mice were subjected to Sham or Doxorubicin treatment. H–N) FGF13 overexpression vector (AAV9‐cTnT‐FGF13) and control vector (AAV9‐LacZ) were injected intravenously into tail veins of 6 weeks old male C57BL/ 6J mice, respectively, 2 weeks after the injection, these mice were subjected to Sham or Doxorubicin treatment. A,H) The experimental flowchart. B,I) Representative M‐mode echocardiographic images from each group in mice and G,N) representative echocardiographic data for LVEF and LVFS are shown (n = 6 per group). C,J) Sirius Red staining (the upper part) (scale bar, 50 µm) and Masson staining (the lower part) (scale bar, 50 µm) and G,N) quantification (n = 6 per group). D,K) Representative image of SA‐*β‐gal* in the heart tissue (Arrows represent positive marks) (scale bar, 50 µm) and G,N) quantification (n = 6 per group). E,L) WGA (wheat germ agglutinin; scale bar, 50 µm) (n = 6 per group) and G,N) quantification (n = 6 per group). F,M) Representative whole heart images (scale bar, 3 mm) and G,N) representative data of HW in the indicated groups (n = 6 per group). Data are means ± SEM.  The P value was determined using ANOVA with Tukey's multiple comparisons test.

Similarly, the myocardial‐specific *Fgf13KO* mice were utilized to further verify the role of FGF13 in the Doxorubicin‐induced premature myocardial aging model (**Figure** **3**A). First, echocardiography showed that upon Doxorubicin exposure, cardiac function, and structure exhibited abnormalities, characterized by decreased ejection fraction and fractional shortening, while myocardial FGF13 deficiency markedly mitigated these outcomes (Figure 3B,G; Table S6, Supporting Information). In addition, the heart appeared shrunken upon Doxorubicin exposure, characterized by a reduction in HW and HW/BW, while myocardial FGF13 deficiency significantly alleviated these effects (Figure 3F,G; Table S6, Supporting Information). Sirius Red and Masson's trichrome staining revealed that the interstitial and perivascular areas of cardiac tissue exhibited a higher degree of fibrosis in Doxorubicin‐exposed murine hearts, while myocardial FGF13 deficiency significantly mitigated this effect (Figure 3C,G; Figure S9A,D, Supporting Information). SA‐*β‐gal* staining showed that myocardial FGF13 deficiency reduced Doxorubicin‐induced premature myocardial aging, as shown by the reduction of SA*‐β‐gal* positive cells (Figure 3D,G). WGA staining revealed that myocardial FGF13 deficiency increased the surface area of cardiomyocytes compared with the Doxorubicin‐treated group (Figure 3E,G). Western blotting demonstrated that myocardial FGF13 deficiency reduced the Doxorubicin‐induced p53 and p21 protein expression (Figure S9B,E,F, Supporting Information). In addition, immunofluorescence revealed that FGF13 deficiency in murine hearts significantly downregulated p21 content in cardiomyocytes upon Doxorubicin exposure (Figure S9C, Supporting Information). It was concluded that myocardial FGF13 deficiency alleviated Doxorubicin‐induced myocardial senescent level and cardiac injury.

As expected, myocardial‐specific FGF13 overexpression exacerbated Doxorubicin‐induced impairment of myocardial function and structure (Figure 3I,N; Table S7, Supporting Information). Compared with the Doxorubicin‐treated group, myocardial‐specific FGF13 overexpression further downregulated HW, HW/BW (Figure 3M,N; Table S7, Supporting Information), and the cardiomyocyte surface area (Figure 3L,N), and elevated the degree of fibrosis (Figure 3J,N; Figure S10A,D, Supporting Information), SA‐*β‐gal* activity (Figure 3K,N). Moreover, FGF13 overexpression further increased the p53 and p21 protein levels (Figure S10B,E,F, Supporting Information), *p21* mRNA level (Figure S18B, Supporting Information), and p21 content of cardiomyocytes (Figure S10C, Supporting Information) upon Doxorubicin treatment. Taken together, these results suggest that myocardial FGF13 deficiency alleviates Doxorubicin‐induced premature myocardial aging and functional impairment.

### 2.4 FGF13 Knockdown Alleviates Premature Cardiomyocyte Senescence

**Figure 4:**
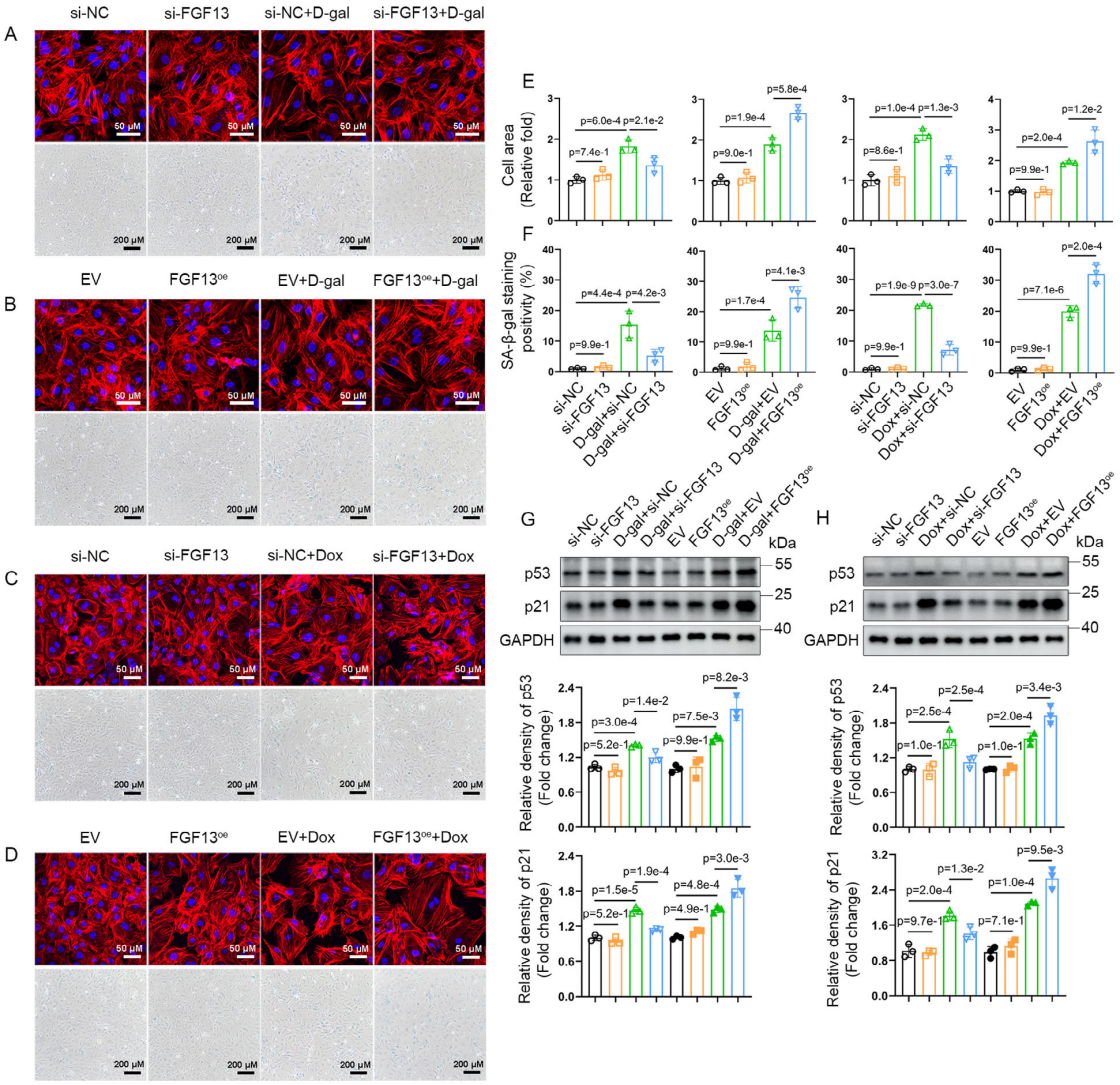
FGF13 knockdown alleviates premature cardiomyocyte senescence. Transfected Neonatal Rat Cardiomyocytes (NRCMs) were either untreated or treated with Doxorubicin (0.1 µm) or D‐galactose (20 g L^−1^) for 72 h and related quantification. A–D) TRITC Phalloidin staining (the upper part) and E) related quantification (n = 3 per group). A–D) *β‐galactosidase* staining (the lower part) and F) related quantification (n = 3 per group). A,C) NRCMs were transfected with si‐FGF13 or si‐NC. B,D) NRCMs were transfected with FGF13^oe^ or empty vector (EV). G,H) Representative western blotting for p53 and p21 in NRCMs and related quantification (n = 3 per group). The protein level was standardized by GAPDH. Data are means ± SEM. The P value was determined using ANOVA with Tukey's multiple comparisons test.

Next, we explored whether FGF13 plays a role in cardiomyocyte senescence. To this end, we constructed two independent small interfering RNAs targeting FGF13, designated as si‐FGF13 and si‐FGF13‐2. Using phalloidin staining technology, we observed that the exposure to D‐galactose and Doxorubicin increased the surface area of cardiomyocytes, while transfection with si‐FGF13 or si‐FGF13‐2, which reduces FGF13 levels (Figure S11A, S12G,J, Supporting Information), could effectively mitigate this effect (**Figure** **4**A,C,E; Figure S12A,B,D,F, Supporting Information). SA‐*β‐gal* staining revealed that FGF13 knockdown alleviated the cardiomyocyte senescent level induced by D‐galactose and Doxorubicin (Figure 4A,C,F; Figure S12A–C,E, Supporting Information). In addition, western blotting showed that treatment with D‐galactose and Doxorubicin significantly increased the expression of p53 and p21 proteins in senescent cardiomyocytes. However, this effect was reversed by the knockdown of FGF13 (Figures 4G,H; S12H,I,K, Supporting Information).

Next, we constructed an FGF13 overexpression plasmid (FGF13^oe^) to further verify the role of FGF13 in cardiomyocyte senescence. As expected, treatment with D‐galactose and Doxorubicin obviously elevated the surface area, SA‐*β‐gal* activity, and the expression of p53 and p21 proteins in cardiomyocytes, while transfection of FGF13^oe^ to overexpress FGF13 (Figure S11B, Supporting Information) further exacerbated these effects. (Figure 4B,D–H). Thus, it was concluded that FGF13 knockdown markedly alleviated premature cardiomyocyte senescence.

### 2.5 FGF13 Regulates the Expression of Cav1 In Vivo and In Vitro in Senescent Cardiomyocytes

**Figure 5:**
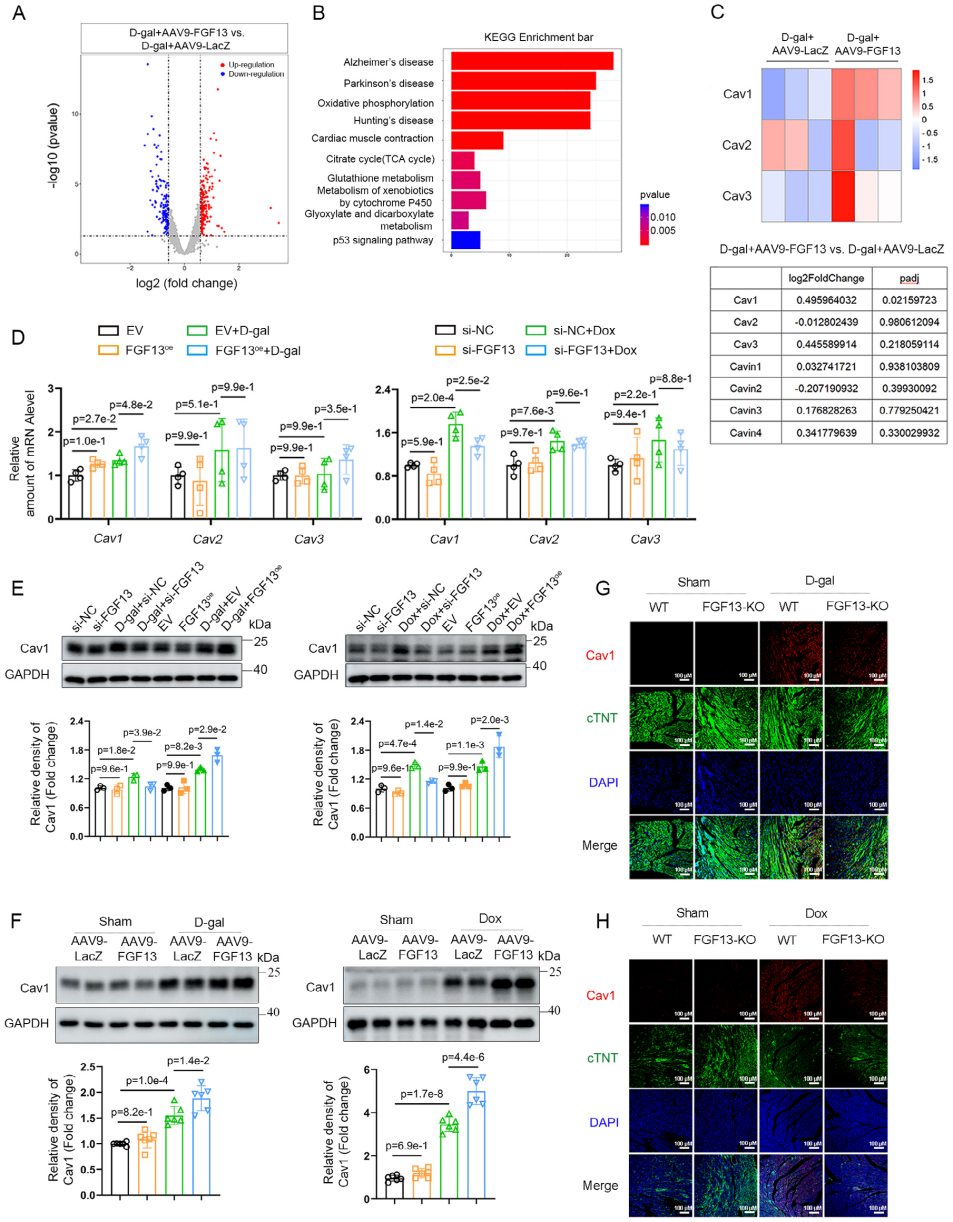
FGF13 regulates the expression of Cav1 in vivo and in vitro in senescent cardiomyocytes. A–C) RNA transcriptome sequencing was performed on D‐galactose‐induced cardiac tissue samples treated with AAV9‐FGF13 (n = 3) and AAV9‐LacZ (n = 3), respectively. A) Volcano plot of differentially expressed genes. We used log2 of the fold change as the source of data for the X axis and‐log10 of the P as the source of data for the y axis. Fold change >1.5× and padj < 0.05 indicate statistically significant differences. Red and blue points represent the upregulated genes and the downregulated genes compared with control group. B) KEGG pathways. The x‐axis is the rich factor. The y‐axis refers to pathway terms. C) The corresponding Cav1‐3 were visualized by the heatmap. The darker shade of red or blue represents the higher correlation level (the upper part). The variation and padj of corresponding Cav1‐3 and Cavin1–4 were shown in the table (the lower part). D) Real‐time qPCR analysis of the *Cav1‐3* mRNA expression in D‐galactose‐induced NRCMs treated with FGF13^oe^ or EV and related quantification (the left part) and in Doxorubicin‐induced NRCMs treated with si‐FGF13 or si‐NC and related quantification (the right part). E) NRCMs were transfected with si‐FGF13 (or si‐NC) and FGF13^oe^ (or EV). Transfected NRCMs were either untreated or treated with D‐galactose or Doxorubicin for 72 h. Representative western blotting results (the upper part) and related quantification of Cav1 in NRCMs (the lower part) (n = 3 per group). F) Representative western blotting results in D‐galactose or Doxorubicin induced murine heart after myocardial‐specific FGF13 overexpression (the upper part) and related quantification of Cav1 (the lower part) (n = 6 per group). G,H) Representative images of immunofluorescence staining of Cav1 (red) and cTNT (green) and DAPI (blue) in D‐galactose and Doxorubicin induced mouse hearts after myocardial‐specific *Fgf13* knockout. Data are means ± SEM. The P value was determined using ANOVA with Tukey's multiple comparisons test.

Next, we explored how FGF13 regulated cardiomyocyte senescence. Comparative gene expression profiling analysis of RNA‐sequencing data for D‐galactose‐exposed murine cardiac tissues treated with AAV9‐FGF13 and AAV9‐LacZ was performed to identify differentially expressed genes by Volcano plot (**Figure** **5**A). KEGG pathway analysis indicated that FGF13 overexpression in the heart was associated with the p53/p21 pathway, which is consistent with our finding that FGF13 overexpression upregulated p53 and p21 in premature aging hearts (Figure 5B). We then proceeded to further explore how FGF13 regulated the p53/p21 pathway. Previous studies showed that in FGF13‐deficient adult mice, Cav3 levels in cardiomyocytes was markedly elevated, protecting against chronic stress‐induced pathology.^[^ ^30^ ^]^ Transcriptomic RNA‐sequencing analysis revealed that myocardial FGF13 overexpression significantly upregulated *Cav1* transcriptional level, while there were no significant differences in the levels of *Cav2‐3* and *Cavin1‐4* (Figure 5C). Studies reported that Cav1 is considered a gatekeeper of cellular senescence, widely used to detect senescent phenotypes,^[^ ^19^, ^20^ ^]^ and can regulate multiple signaling pathways in senescent cells, such as the p53/p21Waf‐1/Cip1 pathway.^[^ ^24^, ^25^ ^]^ In light of this, we further explored whether FGF13 regulates Cav1 expression in premature myocardial aging models. Next, RT‐qPCR revealed that FGF13 overexpression obviously upregulated the *Cav1* mRNA level in D‐galactose‐exposed cardiomyocytes, while there were no significant differences in the levels of *Cav2* and *Cav3*. Similarly, FGF13 knockdown in vitro downregulated the transcriptional activity of *Cav1*, with no significant differences in the levels of *Cav2* and *Cav3* in Doxorubicin‐induced senescent cardiomyocytes (Figure 5D).

Using western blotting technology, we found that FGF13 overexpression and knockdown upregulated and downregulated the Cav1 expression in D‐galactose/Doxorubicin‐treated senescent cardiomyocytes, respectively (Figure 5E). Moreover, myocardial FGF13 overexpression markedly increased the Cav1 protein level in premature aging hearts exposed to D‐galactose and Doxorubicin (Figure 5F). Furthermore, immunofluorescence revealed that myocardial FGF13 deficiency obviously alleviated the content of Cav1 in cardiomyocytes treated with D‐galactose and Doxorubicin (Figure 5G,H). Taken together, these results confirm that FGF13 regulates Cav1 expression in senescent cardiomyocytes in vivo and in vitro.

### 2.6 Myocardial‐Specific Overexpression of Cav1 Reverses the Protective Effect of FGF13 Knockout Against Cardiomyocyte Senescence and Cardiac Injury

**Figure 6:**
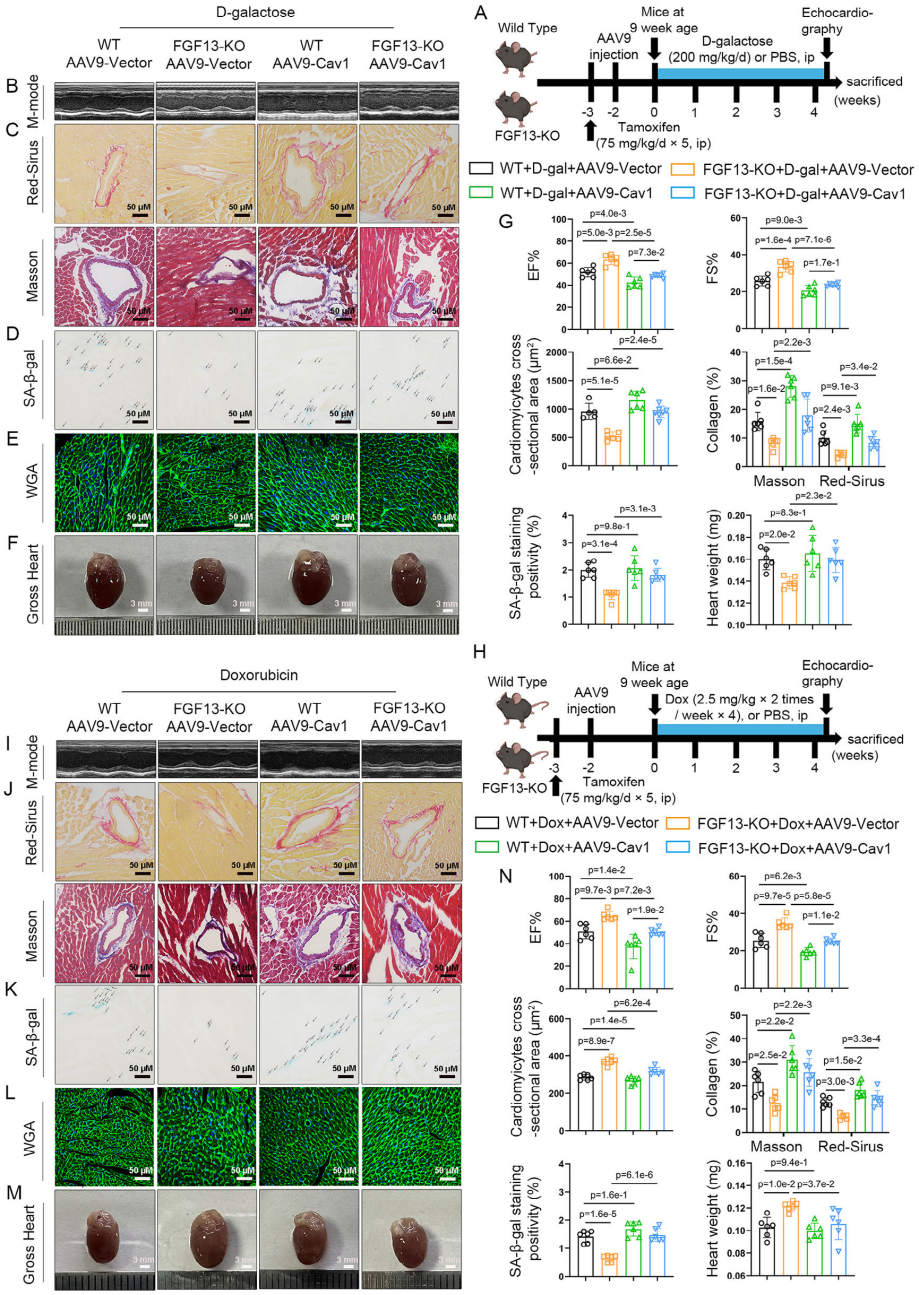
Myocardial‐specific overexpression of Cav1 reverses the protective effect of FGF13 knockout against cardiomyocyte senescence and cardiac injury. For 6 weeks old wild‐type (WT, *Fgf13^f/Y^* ^)^ mice and *Fgf13KO* (*Fgf13^f/Y^* crossed with αMHC‐MerCreMer) mice, tamoxifen was administered at the dose of 75 mg/kg/day for 5 consecutive days. On the 7th week, AAV9‐Cav1 (or AAV9‐LacZ) was injected into the tail vein for 2 weeks. Then these mice were subjected to D‐galactose or Doxorubicin treatment. After successful modeling, cardiac function tests were performed, and tissue samples were collected. A,H) The experimental flowchart. B,I) Representative M‐mode echocardiographic images from each group in mice and G,N) representative echocardiographic data for LVEF and LVFS are shown (n = 6 per group). C,J) Sirius Red staining (the upper part) (scale bar, 50 µm) and Masson staining (the lower part) (scale bar, 50 µm) and G,N) quantification (n = 6 per group). D,K) Representative image of SA*‐β‐gal* in the heart tissue (Arrows represent positive marks) (scale bar, 50 µm) and G,N) quantification (n = 6 per group). E,L) WGA (wheat germ agglutinin; scale bar, 50 µm) (n = 6 per group) and G,N) quantification (n = 6 per group). F,M) Representative whole heart images (scale bar, 3 mm) and G,N) representative data of HW in the indicated groups (n = 6 per group). Data are means ± SEM. The P value was determined using ANOVA with Tukey's multiple comparisons test.

While previous studies have established a relationship among FGF13, Cav1, and the p53‐p21 pathway, the direct mechanisms and their correlations in myocardial premature aging remain unclear. Thereby, we will conduct Cav1 rescue experiments to further elucidate the relationship between FGF13 and Cav1 in myocardial premature aging. Next, we constructed an adeno‐associated virus for Cav1 overexpression (AAV9‐Cav1). Echocardiography results showed that myocardial FGF13 deficiency alleviated D‐galactose‐induced impairment of myocardial function and structure. However, myocardial Cav1 overexpression (Figure S7B, Supporting Information) mitigated these outcomes (**Figure** **6**B,G; Table S8, Supporting Information). In addition, myocardial Cav1 overexpression reversed the inhibitory effects of FGF13 deficiency on D‐galactose‐induced HW and HW/BW (Figure 6F,G; Table S8, Supporting Information), the degree of fibrosis (Figure 6C,G; Figure S13A,D, Supporting Information), SA‐*β‐gal* activity (Figure 6D,G), the cardiomyocyte surface area (Figure 6E,G), the level of p53 and p21 protein (Figure S13B,E,F, Supporting Information), and the p21 content of cardiomyocytes (Figure S13C, Supporting Information). In summary, Cav1 overexpression reverses the protective effect of FGF13 deficiency against D‐galactose‐induced myocardial aging and cardiac injury.

**Figure 7:**
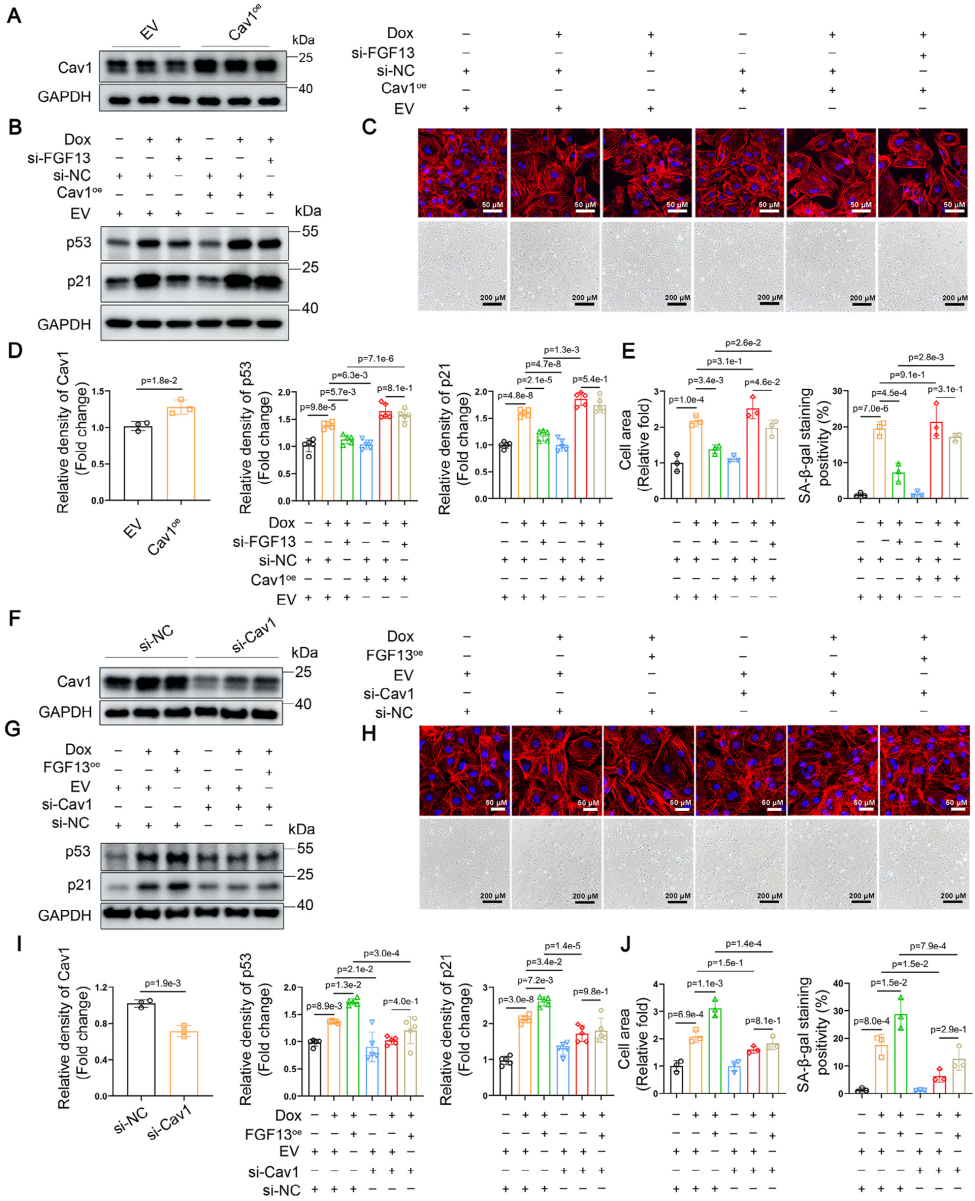
The regulation of premature senescence in cardiomyocytes by FGF13 in vitro depends on the expression of Cav1. NRCMs were either untreated or treated with Doxorubicin (0.1 µm) for 72 h. A–E) NRCMs were transfected with si‐FGF13 (si‐NC) and Cav1^oe^ (or EV). F–J) NRCMs were transfected with FGF13^oe^ (or EV) and si‐Cav1 (or si‐NC). B,G) Representative western blotting results and D,I) related quantification of p53, p21 (n = 5 per group). A,F) NRCMs were transfected with Cav1^oe^ (or EV) and si‐Cav1 (or si‐NC). Representative Western blotting results for Cav1 and D,I) related quantification (n = 3 per group). The protein level was standardized by GAPDH. C,H) TRITC Phalloidin staining (the upper part) and *β‐galactosidase* staining (the lower part) and E,J) related quantification (n = 3 per group). Data are means ± SEM. The P value was determined using two‐tailed unpaired Student's t test or ANOVA with Tukey's multiple comparisons test.

Consistently, we obtained similar results in Doxorubicin‐induced premature aging hearts.

Myocardial Cav1 overexpression reversed the inhibitory effects of FGF13 deficiency on Doxorubicin‐induced myocardial impaired function and structure (Figure 6I,N; Table S9, Supporting Information), the degree of fibrosis (Figure 6J,N; Figure S14A,D, Supporting Information), SA‐*β‐gal* activity (Figure 6K,N), the p53 and p21 protein levels (Figure S14B,E,F, Supporting Information), and the p21 content of cardiomyocytes (Figure S14C, Supporting Information) and alleviated the positive effects of FGF13 deficiency on the HW and HW/BW (Figure 6M,N; Table S9, Supporting Information), cardiomyocyte surface area (Figure 6L,N). However, myocardial‐specific Cav1 overexpression did not increase D‐galactose/Doxorubicin‐induced premature myocardial aging and cardiac injury, as indicated by HW and HW/BW, the cardiomyocyte surface area, and SA‐*β‐gal* activity (Figure 6). Moreover, we found that myocardial‐specific Cav1 overexpression alone is not sufficient to alter myocardial function and structure compared to the Sham group (Figure S15F,G; Table S10, Supporting Information). It was detected that myocardial specific Cav1 overexpression did not markedly elevate the level of HW and HW/BW (Figure S15A,I; Table S10, Supporting Information), the degree of fibrosis (Figure S15D,E,K, Supporting Information), SA‐*β‐gal* activity (Figure S15B,J, Supporting Information), and the cardiomyocyte surface area (Figure S15C,H, Supporting Information). Taken together, myocardial‐specific overexpression of Cav1 reverses the protective effect of FGF13 knockout against cardiomyocyte senescence and cardiac injury.

### 2.7 FGF13 Modulates Cardiomyocyte Premature Senescence Through Cav1 Regulation In Vitro

Next, we explored whether the regulation of cardiomyocyte senescence by FGF13 is modulating Cav1 expression in vitro. Cav1 rescue experiments was used to detect that Cav1 overexpression (**Figure** **7**A,D) reversed the alleviatory effect of FGF13 knockdown on the Doxorubicin‐induced p53 and p21 protein levels (Figure 7B,D), the content of p21 in cardiomyocytes (Figure S16, Supporting Information), the surface area of cardiomyocytes and SA‐*β‐gal* activity (Figure 7C,E). Consistently, we obtained similar results in D‐galactose‐induced senescent cardiomyocytes (Figure S17, Supporting Information).

Next, we constructed small interfering RNA targeting Cav1 (si‐Cav1). Upon treatment with Doxorubicin, FGF13 overexpression in cardiomyocytes further increased the protein levels of p53 and p21 (Figure 7G,I), the surface area of cardiomyocytes, and SA‐*β‐gal* activity (Figure 7H,J), while transfection with si‐Cav1, which reduced Cav1 levels (Figure 7F,I), could reverse these effects. Moreover, it was found that Cav1 knockdown could downregulate Doxorubicin‐induced p53, p21 protein levels, the surface area, and cardiomyocyte senescent level (Figure 7F–J). Taken together, these findings demonstrate that the regulation of premature senescence in cardiomyocytes by FGF13 in vitro depends on the expression of Cav1.

### 2.8 FGF13 Regulates Cav1 Activity and Its Upstream Pathway to Participate in Cardiomyocyte Senescence

**Figure 8:**
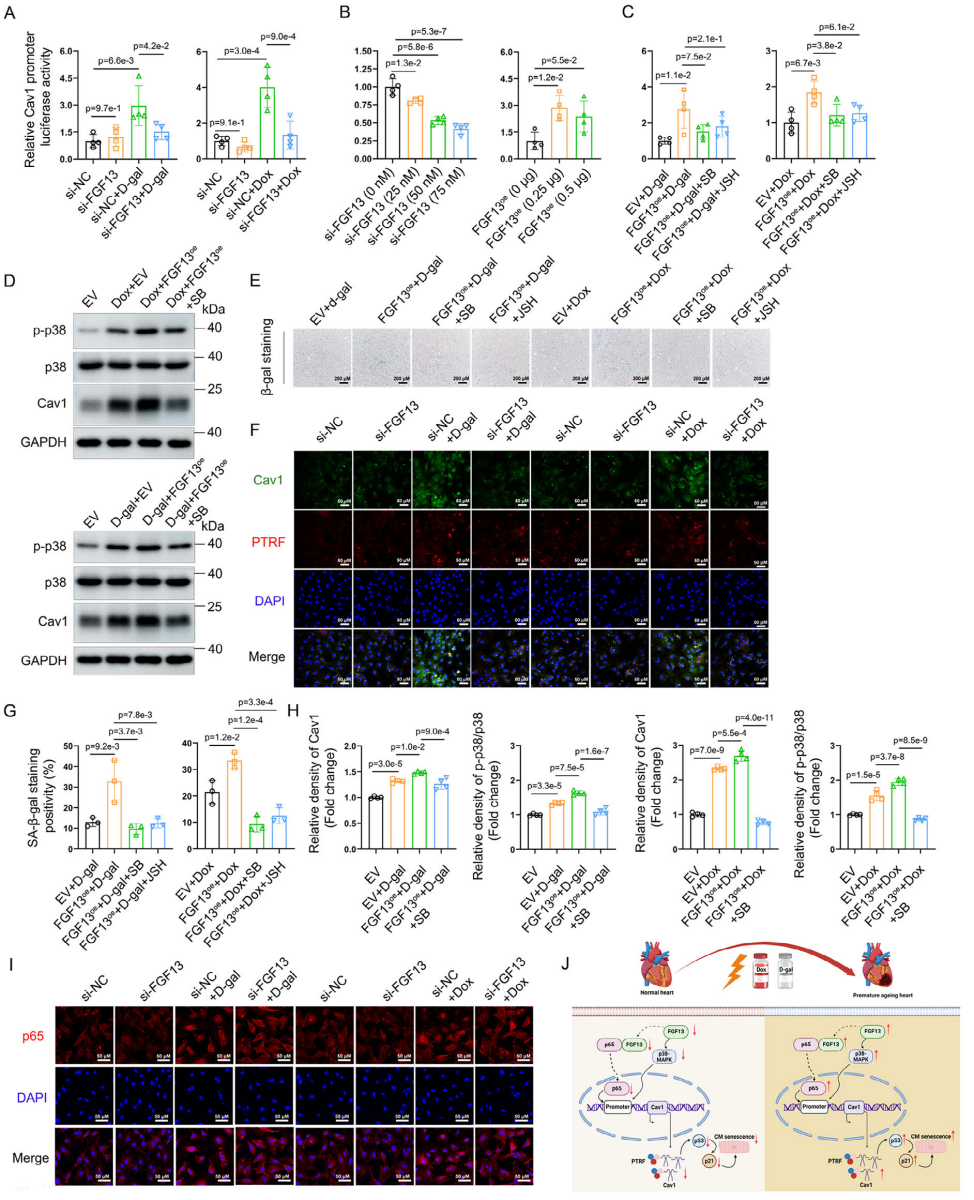
FGF13 regulates Cav1 activity and its upstream pathway to participate in the cardiomyocyte senescent process. NRCMs transfected with si‐FGF13 (or si‐NC) or FGF13^oe^ (or EV), in the presence or absence of JSH‐23 (20 µm) or SB203580 (20 µm) for 3 h, and then were either untreated or treated with Doxorubicin (0.1 µm) or D‐galactose (20 g L^−1^) for F,I) 24 h, A,C) 48 h, D,E) 72 h. HEK293T cells were transfected with B) si‐FGF13 (0, 25, 50, 75 nm) or FGF13^oe^ (0.25, 0.5 µg) for 48 h (n = 4 per group). A–C) Luciferase reporter activities of Cav1. D) Representative western blotting results and related quantification of H) Cav1, and the ratio of p‐p38 to p38 (n = 4 per group). The protein level was standardized by GAPDH. E) *β‐galactosidase* staining (n = 3 per group) and G) related quantification. F) Representative images of immunofluorescence staining of Cav1 (green) and PTRF (red) and DAPI (blue) (scale bar, 50 µm) (n = 3 per group). I) Representative images of immunofluorescence staining of p65 (red) and DAPI (blue) (scale bar, 50 µm) (n = 3 per group). J) The graphic abstract. Created in BioRender. shen, m. (2025) https://BioRender.com/0ul3s50. Data are means ± SEM. The P value was determined using ANOVA with Tukey's multiple comparisons test.

Next, we explored how FGF13 regulated the expression of Cav1. As previously revealed, FGF13 overexpression upregulated the *Cav1* transcriptional level in D‐galactose‐induced cardiac tissues (Figure 5C). Meanwhile, FGF13 is positively correlated with *Cav1* transcriptional activity in senescent cardiomyocytes (Figure 5D). Recent studies showed that the expression of Cav1 can be regulated by p38‐MAPK,^[^ ^21^ ^]^ Sp1,^[^ ^22^ ^]^ NF‐κB^[^ ^23^ ^]^ in senescent cells. Therefore, we investigated whether FGF13 modulates Cav1 through these mechanisms to participate in cardiomyocyte senescence. To this end, we constructed a Cav1 promoter construct. A luciferase reporter assay revealed that FGF13 knockdown markedly reduced Cav1 promoter activity in cardiomyocytes treated with D‐galactose and Doxorubicin (**Figure** **8**A). Similarly, FGF13 expression positively correlated with Cav1 promoter activity in 293T cells (Figure 8B).

Subsequently, we pretreated cardiomyocytes with SB203580, an inhibitor of p38‐MAPK, or JSH‐23, an inhibitor of NF‐κB. A luciferase reporter assay revealed that inhibition of the p38‐MAPK pathway or the NF‐κB pathway obviously downregulated the Cav1 promoter activity induced by FGF13 overexpression in senescent cardiomyocytes (Figure 8C). Western blotting revealed that pretreatment with SB203580 reversed the upregulating effect of Cav1 protein expression by FGF13 overexpression, and simultaneously suppressed the ratio of phospho‐p38 to total p38 (Figure 8D,H).

Moreover, SA‐*β‐gal* staining revealed that treatment with SB203580 or JSH‐23 reversed the stimulatory effect of FGF13 overexpression on D‐galactose/Doxorubicin‐induced cardiomyocyte senescence, as evidenced by a decrease in the number of senescent positive staining cells (Figure 8E,G). Furthermore, immunofluorescence revealed that FGF13 knockdown decreased D‐galactose/Doxorubicin‐induced nuclear translocation of p65 in cardiomyocytes (Figure 8I). Recent studies showed that PTRF can directly interact with Cav1 and participate in downstream pathways such as the p53‐p21 pathway.^[^ ^36^, ^37^ ^]^ FGF13 overexpression enhanced binding of Cav1 to PTRF in cardiomyocytes treated with D‐galactose or Doxorubicin (Figure 8F).

Taken together, it is concluded that FGF13 regulates Cav1 promoter activity through the p38/MAPK pathway and nuclear translocation of p65, and mediates p53‐p21 pathway to regulate cardiomyocyte senescence by upregulating the binding levels of PTRF to Cav1.

## 3 Discussion

Cellular senescence, which occurs in aging individuals but also across the lifespan in association with tissue damage, acute and chronic diseases.^[^ ^38^ ^]^ In addition to replicative senescence, the senescent state can also be induced by stressors, known as premature senescence.^[^ ^3^ ^]^ Recently, emerging evidence has revealed a connection between cardiomyocyte senescence and CVDs.^[^ ^4^, ^5^, ^6^ ^]^ Cardiomyocyte senescence is typically characterized by enlarged cell size, DNA damage responses, SA‐*β‐gal* activity, the senescence‐associated secretory phenotype, and upregulation of senescent protein markers, including p53 and p21.^[^ ^39^ ^]^ Suppressing cardiomyocyte senescence not merely reduces aging‐associated inflammation but also affects other myocardial lineages, implying a broader propagation mechanism in pathological remodeling.^[^ ^14^, ^40^ ^]^

Recent studies have targeted cardiomyocyte senescence to alleviate adverse remodeling after ischemia‐reperfusion.^[^ ^4^ ^]^ Studies also showed that circHIPK3 deficiency exacerbated cardiomyocyte senescence, thereby damaging heart function and promoting cardiac aging.^[^ ^5^ ^]^ Other studies showed that the SIRT2‐STAT3‐CDKN2B pathway is crucial in the senescence of primate cardiomyocytes and has a potential to reverse cardiac aging.^[^ ^6^ ^]^ Thus, it can be seen that understanding the mechanisms by which cardiomyocyte senescence induces CVDs is crucial for the development of related therapeutic strategies.

FGFs comprise a multifunctional family of polypeptide growth factors with at least 22 distinct members.^[^ ^28^ ^]^ The roles of FGFs and their receptors in aging and aging‐related diseases are gradually being revealed. For example, *Fgf23*‐knockout mice exhibit an accelerated senescence‐like phenotype^[^ ^41^ ^]^ and FGF ligands and receptors are downregulated in aging tissues such as brain, bone, and skin.^[^ ^42^ ^]^ In addition, studies revealed that FGF21 improves inflammatory response, indirectly delays cellular senescence, and directly plays an anti‐aging role by activating autophagy genes^[^ ^43^ ^]^ Initially, we were interested in exploring the potential roles of lesser‐studied FGF family members, beyond the well‐known FGF21 and FGF23, in cardiac aging and heart failure. By analyzing the GSE12480 and GSE56348 datasets, we discovered that FGF13 was the most significantly and consistently downregulated FGF member in both aged and failing hearts compared to controls. This finding led us to investigate FGF13's role in premature myocardial senescence through in vivo and in vitro experiments. While our results emphasize FGF13's importance, we acknowledge that other senescence‐associated genes may also contribute to this complex biological phenomenon, warranting further investigation in future studies.

FGF13 reportedly participates in many pathophysiological processes, including cardiac dysfunction.^[^ ^29^, ^30^ ^]^ However, whether FGF13 is associated with cardiomyocyte senescence and premature cardiac aging remains unclear. In our study, upon long‐term exposure to Doxorubicin or D‐galactose, the murine heart exhibited premature aging and downregulation of FGF13 expression. In vivo, myocardial‐specific knockout and overexpression of FGF13 alleviated and exacerbated Doxorubicin/D‐galactose‐induced myocardial dysfunction, fibrosis, and aging characteristics, respectively. In vitro, FGF13 knockdown and overexpression alleviated and aggravated the cardiomyocyte senescent level, accompanied by decreasing or increasing the cardiomyocyte surface area, *β‐galactosidase* activity, and protein levels of the aging markers p53 and p21.

We next explored how FGF13 regulated premature cardiomyocyte aging. RNA‐sequencing revealed that FGF13 was associated with Cav1 activity and the p53‐p21 pathway. Cav (Caveolin) is the principal protein component of Caveolae, with three subtypes, namely Cav1, 2, and 3.^[^ ^17^ ^]^ Among Cav family members, Cav1 is considered a gatekeeper of cellular aging and is widely used to detect aging phenotypes.^[^ ^18^, ^19^ ^]^ qRT‐PCR showed that in FGF13 mediated premature cardiac aging induced by Doxorubicin and D‐galactose, the change of Cav1 was more significant than those of Cav2 and Cav3. These studies have linked FGF13, Cav1, and the p53‐p21 pathway, but their roles in myocardial premature aging are unclear. Next, we will conduct Cav1 rescue experiments to clarify the relationship between FGF13 and Cav1 in this context. Thereby, first, we conducted in vivo experiments to verify the role of Cav1 in regulation of premature myocardial aging by FGF13. We also constructed Cav^oe^ and si‐Cav1 to verify the dependency of FGF13‐mediated regulation of premature cardiomyocyte senescence on Cav1 in vitro.

We then explored how FGF13 regulates Cav1 activity in premature myocardial aging models. Considering that the expression of Cav1 in senescent cells is regulated by p38‐MAPK,^[^ ^21^ ^]^ Sp1,^[^ ^22^ ^]^ NF‐κB^[^ ^23^ ^]^ and Cav1 regulates several signaling pathways in senescent cells, including p53/p21Waf‐1/Cip1 pathway.^[^ ^24^, ^25^ ^]^ Thus, we investigated the related upstream pathways of Cav1 and found that FGF13 regulated the transcriptional activity and expression of Cav1 through the p38/MAPK pathway and nuclear translocation of p65, and mediated p53‐p21 pathway to regulate cardiomyocyte senescence by upregulating the binding levels of PTRF with Cav1.

However, our study still has some limitations. First, we demonstrated that FGF13 has a role in premature myocardial aging, but did not investigate the roles of any other family members. For example, we found that cardiomyocyte‐specific knockout of FGF13 affected the gene and protein levels of certain FGF family members, such as FGF6 (Figure S5, Supporting Information). However, whether these changes participate in the regulation of myocardial premature aging or cardiomyocyte senescence by FGF13 warrants further in‐depth investigation. Second, we performed long‐term injection of D‐galactose and used multiple low doses of Doxorubicin to induce mouse models of premature myocardial aging and explored the role of FGF13 through Cav1 and the p53‐p21 pathway, but did not perform further validation in myocardial tissue of naturally aging mice. While our database analysis revealed a significant reduction in FGF13 levels in aging human myocardial tissues relative to younger controls—a finding corroborated by our observations in both naturally aged mice and D‐galactose/Doxorubicin‐induced premature myocardial aging models—we recognize that the modest sample size of clinical specimens constrains the study. This limitation underscores the necessity for broader clinical studies to validate and expand upon these insights in subsequent research endeavors. Additionally, further investigation is warranted to uncover additional signaling pathways that may be implicated in FGF13's regulation of cardiomyocyte senescence.

In conclusion, our results demonstrate that the murine myocardium exhibites premature aging characteristics upon exposure to D‐galactose and Doxorubicin, accompanied by significant downregulation of FGF13. Myocardial‐specific knockout and overexpression of FGF13 alleviated and exacerbated D‐galactose/Doxorubicin‐induced cardiac dysfunction, myocardial fibrosis, and aging characteristics in vivo, respectively. Mechanistically, FGF13 regulated Cav1 promoter activity and expression through the p38/MAPK pathway and nuclear entry of p65, and mediated activation of the p53‐p21 pathway to increase cardiomyocyte senescence by increasing binding of PTRF to Cav1 (Figure 8J). In summary, we demonstrated that FGF13 regulates the Cav1‐p53‐p21 axis to augment premature cardiomyocyte senescence and exacerbate premature cardiac aging.

## 4 Experimental Section

Detailed reagent information, cell culture treatment, and experimental methods are described in the Supporting Information in the online supplementary file. Primer sequences for PCR analysis and sequences of gRNA were shown in Tables. The data, analytical methods, and study materials are available from the corresponding author on reasonable request.

## Conflict of Interest

The authors declare no conflict of interest.

## Author Contributions

E.Z.S., Y.C.W., and W.J.Y. contributed equally to this work. W.T.C., X.W., K.Y.L., L.T.J., and X.K.L. contributed to the literature search and study design. E.Z.S., W.J.Y., and S.H.L. participated in the drafting of the article. E.Z.S., Y.C.W., W.J.Y., J.J.Z., G.Y.C., and M.F.J. carried out the experiments. E.Z.S., W.J.Y., Y.C.W., Z.C.H., W.J.Y., X.J.Y., and Z.H.T. revised the article. E.Z.S., Y.C.W., G.Y.C., X.J.Y., and Z.H.T. contributed to data collection and analysis. All authors reviewed the manuscript.

## Supporting information



Supporting Information

## Data Availability

The data that support the findings of this study are available on request from the corresponding author. The data are not publicly available due to privacy or ethical restrictions.
