# Supplementary material for: The FGF13‐Caveolin‐1 Axis: A Key Player in the Pathogenesis of Doxorubicin‐ and D‐Galactose‐Induced Premature Cardiac Aging
Source: Adv Sci (Weinh). 2025 Apr 4;12(25):2501055. doi: 10.1002/advs.202501055 (PMC12224973; doi:10.1002/advs.202501055)
Supplement: Supplementary file 1 — Supporting Information [file ADVS-12-2501055-s001.docx]

**Supporting Information**

**The FGF13-Caveolin-1 axis: A Key Player in the Pathogenesis of Doxorubicin- and D-galactose-induced Premature Cardiac Aging**

***Authors***

*Enzhao Shen, Yuecheng Wu, Weijian Ye, Sihang Li, Junjie Zhu, Meifan Jiang, Zhicheng Hu, Gaoyong Cao, Xiaojing Yi, Fan Li, Zhouhao Tang, Xiaokun Li, Kwang Youl Lee, Litai Jin****^*^****, Xu Wang****^*^****, Weitao Cong****^*^.***

**AFFILIATIONS**

E. Shen, Y. Wu, S. Li, J. Zhu, M. Jiang, Z, Hu, G. Cao, X. Yi, Z. Tang, X. Li, L. Jin, X. Wang, W. Cong**.**

**School of Pharmaceutical Science, Wenzhou Medical University, Wenzhou 325035, China**

**E-mail:** [jin_litai@126.com](mailto:jin_litai@126.com)**; wang_xu2003@wmu.edu.cn;** [cwt97126@126.com](mailto:cwt97126@126.com)

E. Shen, K. Lee

College of Pharmacy, Research Institute of Pharmaceutical Sciences, Chonnam National University, Gwangju 61186, Republic of Korea

J. Zhu, M. Jiang, Z, Hu, F. Li, Z. Tang, X. Li, W. Cong**.**

**Oujiang Laboratory (Zhejiang Lab for Regenerative Medicine, Vision and Brain Health), School of Pharmaceutical Science, Wenzhou Medical University, Wenzhou, P.R. China.**

L. Jin

**Ningbo Key Laboratory of Skin Science, Ningbo College of Health Sciences, Ningbo, 315000, P.R. China**

W. Ye

Department of Pharmacy, The Second Affiliated Hospital and Yuying Children's Hospital of Wenzhou Medical University, Wenzhou, 325027, PR China

**This file includes the following subsections:**

**Supplementary Materials and Methods Regents;**

**Figure S1-S18;**

**Table S1-S10.**

**Supplementary Materials and Methods Regents;**

**Animal procedures**

To ensure compliance with ethical research standards, our study obtained approval from the Institutional Animal Care and Use Committee of Wenzhou Medical University, China. The adult male C57BL/6J mice, aged six weeks, were provided by the Model Animal Research Center in Nanjing University, China. The young (3-4 months) and old (24-26 months) C57BL/6J male mice, and *Fgf13 ^flox/flox^* (*Fgf13^f/f^*) or *Fgf13 ^flox/Y^* (*Fgf13 ^f/Y^*) mice on C57BL/6J background were generously provided by Professor Cong of Wenzhou University.

To evaluate the roles of FGF13 on cardiac function in vivo, we generated inducible, cardiac-specific Fgf13 knockout mice. The murine *Fgf13* gene is located on the X chromosome, and spans approximately 72.3 kb. Mice with tamoxifen-inducible Cre-fusion protein under the control of the cardiomyocyte-specific α-myosin heavy-chain promoter (αMHC-Mer CreMer) were previously described^1^. *Fgf13^-/Y^* (*Fgf13^f/Y^*; αMHC-MerCreMer) and *Fgf13^-/-^* (*Fgf13^f/f^;* αMHC-MerCreMer), which are collectively denoted “knockout” (Fgf13 KO) mice (Figure S4).

For wild-type (*Fgf13^f/f^* and *Fgf13^f/Y^*) mice and *Fgf13*-KO (*Fgf13^f/f^* and *Fgf13^f/Y^* crossed with αMHC-MerCreMer) mice, tamoxifen (Santa Cruz Biotechnology, sc-208414) was administered at the dose of 75 mg/kg/day for 5 consecutive days by intraperitoneal (i.p) injection. Genotypes of the transgenic mice were detected by polymerase chain reaction (PCR) analysis using the DNA from the mouse tail, and the specific primers were used as follows:

αMHC-MerCreMer

Forward: 5’-GCGGTCTGGCAGTAAAAACTATC-3’

Reverse: 5’-GTGAAACAGCATTGCTGTCACTT-3’

*Fgf13f/f*

Forward: 5’-AGCGCTCTAAAATTCAGGATGC-3’

Reverse: 5’-CTTCAGGAATAAATGGGGACAGAT-3’

Breeding of transgenic mice follows Mendel’s laws, ensuring a balanced sex ratio. Prior to the experiments, all mice were acclimated in an environmentally controlled room for one week. All mice, free access to water and food, were maintained under standard laboratory conditions with a temperature of 21 ± 2 °C, a relative humidity of 50 ± 15%, and 12 h light-dark cycles. Upon reaching an experimental endpoint, all mice were euthanized by cervical dislocation.

AAV9 harboring FGF13 overexpression vector (pAAV-cTNT-EGFR-P2A-Fgf13-3xFLAG-WPRE) (5 × 10^12^ vg/ml, OBIO technology (Shanghai), reference sequence of Fgf13: NM_010200.3 ) and control vector (pAAV-cTNT-EGFR-P2A-3xFLAG-WPRE) (5 × 10^12^ vg/ml, OBIO technology (Shanghai)); Cav1 overexpression vector (pAAV-cTNT-EGFR-P2A-Cav1-3xFLAG-WPRE) (5 × 10^12^ vg/ml, OBIO technology (Shanghai), reference sequence of Cav1: NM_007616.4) and control vector (pAAV-cTNT-EGFR-P2A-3xFLAG-WPRE) (5 × 10^12^ vg/ml, OBIO technology (Shanghai)), were administered intravenously through the tail vein to male C57BL/6J mice at 6 or 7 weeks of age. 2 weeks later, these mice were intraperitoneal injection of D-galactose at a dosage of 200 mg/kg daily for a period of 8 weeks,^[2,3]^ along with Sham group that received an equal volume of PBS (phosphate buffer saline) injections, or Doxorubicin (2.5 mg/kg/time × 2 per week for 4 consecutive weeks)^[4,5]^ and an equal volume of PBS injections. Upon reaching an experimental endpoint, echocardiography was carried out, and tissue samples were harvested for subsequent analysis.

**Cell Culture and Transfection**

Neonatal rat cardiomyocytes (NRCMs) were isolated according to previously described standard protocols^6,7^. Briefly, neonatal rat born within three days were sacrificed and their hearts were excised and cleaned with cold D-PBS to remove blood. The tissue was minced and placed in a trypsin solution at 37 °C with periodic magnetic stirring at 100 rpm for 8-minute periods. After digestion, cardiomyocytes were removed from fibroblasts using differential adhesion. These cells were then plated on collagen-coated wells and cultured in DMEM/F-12 (Gibco, 11330032) with 10% fetal calf serum (Gibo, 16010159) and 1% penicillin/streptomycin, incubated at 37 °C with 5% CO_2_ for future experiments.

Then NRCMs were transfected with overexpression plasmids for FGF13 (EV, FGF13^oe^) and Cav1 (EV, Cav1^oe^) and small interfering RNAs for FGF13 (si-NC, si-FGF13) and Cav1 (si-NC, si-Cav1), by Lipofectamine 2000 (Invitrogen, cat. no. 11668-019) in Opti-MEM (Gibco, cat. no. 51985034) for 8-12 h. After the transfection, cells were removed to a full-growth medium for another 12 h. Pretreatment with SB203580 (20 μM, MedChemExpress, HY-10256), an inhibitor of p38 MAPK or JSH-23 (10 μM, Selleck, 749886-87-1), an inhibitor of NF-κB transcriptional activity for 3 h, then primary cardiomyocytes or fibroblasts were incubated with Doxorubicin (0.1 μM, MCE, HY-15142) or D-galactose (20 g/L, Nanjing Xinfan, XF8352) for different times to induce cardiomyocyte senescence.

HEK293T cells (cat no. SCSP-502), procured from the Shanghai Institute of Biochemistry and Cell Biology (Shanghai, China), were cultured in DMEM with 4.5 g/L glucose, supplemented with 10% FBS and 1% penicillin/streptomycin, under 5% CO_2_ at 37 °C. Upon reaching 60% confluence, the cells were transfected for 6-8 h and then removed to a full-growth medium for another 48 h for subsequent experiments.

**Echocardiography**

Echo assessments were conducted using a Vevo 1100 Ultrasound System (VisualSonics, Toronto, Canada) equipped with a high-frequency (30 MHz) linear array transducer. Mice were anaesthetized with 3% isoflurane, maintaining heart rates of 400-500 bpm. Chest hair was removed, and body temperature was kept at approximately 37 °C with a heat pad and rectal probe. Systolic and diastolic anatomic parameters were obtained from M-mode tracings at the mid-papillary level, with images stored as cine loops. Subsequent analysis was performed off-line on a workstation installed with Vevo LAB software (version 1.7.1) (VisualSonics, Toronto, Canada).

**Western Blotting**

Briefly, after measuring the protein concentration using the BCA method, 30 μg protein from each sample was resolved by SDS-PAGE on Tris-glycine gels, and transferred to a polyvinylidene fluoride membrane. Membranes were blocked with 5% bovine serum albumin (BSA, Sigma-Aldrich, B2064) in Tris-buffered saline (Sigma-Aldrich, T5030) containing 0.1% Tween 20 (Sigma-Aldrich, 93773) (TBST) and incubated with primary antibodies overnight at 4 °C. Membranes were washed 3 times for 5 min with TBST. This was followed by incubation with HRP-goat-anti-mouse (Abcam, cat. no. ab205719) or HRP-goat-anti-rabbit (Abcam, cat. no. ab205718) secondary antibodies (1h, room temperature). Bound antibody was visualized using a Chemiluminescent HRP Substrate (Millipore, cat. no. WBKLS0500). The protein bands were analyzed by exposure machine (GE, Amersham 154 Imager680) and quantified using Image Quant 5.2 software (Molecular Dynamics, Sunnyvale, CA). The primary antibodies were FGF13 (Affinity biosciences, DF4699, 1:1000), p21 ( ABclonal, A19094, 1:1000 ), p21 ( Santa cruz, sc-6246, 1:1000), p53 (CST, 2524S, 1:1000), Caveolin-1 (Proteintech, 16447-1-AP, 1:1000), p-p38 (ABclonal, AP0526, 1:1000), p38 (ABclonal, A4771, 1:1000), p65 (HUBIO, ET1603-12, 1:1000), FGF1 (HUBIO, HA722695, 1:1000), FGF5 (HUBIO, ER1908-83, 1:1000), FGF6 (ABclonal, A7672, 1:1000), FGF7 (Santa cruz, sc-365440, 1:500), FGF8 (Affinity biosciences, DF2497, 1:1000), FGF9 (Santa cruz, sc-373716, 1:500), FGF11 (HUBIO, ER65621, 1:1000), FGF12 (ABclonal, A2667, 1:1000), FGF14 (HUBIO, ER61580, 1:1000), FGF16 (Santa cruz, sc-390547, 1:500), FGF18 (HUBIO, ER60001, 1:1000), FGF20 (Affinity biosciences, DF8946, 1:1000), FGF21 (Abcam, ab171941, 1:1000).The expression of GAPDH (glyceraldehyde-3-phosphate dehydrogenase) (HUBIO, ET1601-4, 1:5000) was used as a loading control.

**RNA isolation and quantitative real-time-PCR (qRT-PCR)**

Total RNA was extracted from cardiomyocytes and heart tissue using TRIzol reagent (Sigma, T9424), as described by the manufacturer’s instructions. After measuring the total RNA concentration in each sample, the cDNA was synthesized from l μg of RNA according to the manufacturer’s instructions of iScript cDNA Synthesis kit (Bio-Rad, 1708890). Then, qRT-PCR was used to amplify cDNA from samples in each group with specific primers. The relative gene expression levels were calculated using the 2^−ΔΔCt^ method and normalized to the amount of endogenous Glyceraldehyde-3-phosphate dehydrogenase (GAPDH). The sequences of specific primers used for qRT-PCR in this study are listed in Table S1.

**Heart weight, body-weight assessment, and histological examination**

Firstly, the animals were euthanized, and their hearts were collected, fixed in diastole using a 0.5% KCl solution in PBS. Heart weights were recorded and indexed to body weight. Then, part of each heart was immersed in a 4% paraformaldehyde perfusion at 4 °C overnight. Subsequently, the tissue samples were transferred to 70-100% ethanol for dehydration prior to paraffin embedding. Heart sections (5 µm intervals) were deparaffinized and stained with picro sirius red staining (solarbio, S8060), Masson staining (G1340), following standard protocols. Images were acquired using Nikon Eclipse Ni light microscopy.

**Wheat germ agglutinin staining**

Heart sections were incubated with wheat germ agglutinin-Alexa488 (WGA-Alexa488, Solarbio, I3300) for 1 h to visualize the membranes and with 4,6 diamidino-2-phenylindole (DAPI) for 30 min to observe the nuclei. ImagePro Plus software version 7.0 (Media Cybernetics, Rockville, MD) was utilized for all morphometric analyses in a blinded fashion. Sections were measured using a Leica TCS SP8 Confocal microscope (Leica, Wetzlar, Germany).

**SA-*β-gal* staining of tissue and cells**

SA-*β-gal* staining was performed following the manufacturer's instructions (Senescence *β-Galactosidase* Staining Kit, C0602, BEYOTIME). The samples were fixed in 4% paraformaldehyde for 15 min and then rinsed with PBS for 3 times. Then, the fixed sections or cells were incubated with reaction buffer overnight at 37 °C without CO_2_. The sections were counterstained with eosin for 5 min and then dehydrated. Quantification of the SA-*β-gal*-positive area was performed in random cortical images by counting the percentage of positively stained areas in each microscopic field.

**Luciferase Assay**

HEK293T cells or primary cardiomyocytes seeded in 24-well plates were transfected with CMV promoter-driven β-galactosidase (pCMV-β-gal) plasmids in combination with the indicated Caveolin-1 luciferase reporter plasmids (OBIO technology (Shanghai), reference sequence of Cav1 promoter: NM_031556.3). Luciferase Assay System (Promega, Madison, WI, USA) was used to measure the luciferase activity of indicated cell groups following the manufacturer’s protocol, and the corresponding β-gal activity was normalized to determine transfection efficiency in each group. All experiments were performed in at least triplicate.

**TRITC-Phalloidin Staining**

Cardiomyocytes, plated in glass-bottom dishes, cleaned twice with sterile PBS, and then fixed with 4% formaldehyde solution for ten minutes at room temperature. After the excess formaldehyde was removed, the cells were permeated with 0.5 % Triton X-100 solution for five minutes. Then, cardiomyocytes were incubated with Fluor TM 647 phalloidin working solution (YEASEN, 40762ES75) at room temperature in the dark for thirty minutes. Finally, the nuclei were stained with DAPI solution, followed by fluorescence observation under a fluorescence microscope.

**Immunofluorescent staining**

Briefly, the cardiomyocytes and deparaffinized heart sections (5 µm intervals) were subsequently fixed with 4% paraformaldehyde for 20 min, followed by permeabilization using 0.5% Triton X-100 in PBS for 20 min. After wash, the samples were blocked with 5% BSA in PBS for 30 min prior to overnight incubation with the primary antibody at 4 °C. In our studies, the following primary antibodies were used: CTNT (ABclonal, A4914, 1: 200), CTNT (HUBIO, EM1701-39, 1:500), Vimentin (HUBIO, EM0401, 1:200), CD31 (Abcam, ab28364 ,1:200), FGF13 (Affinity biosciences, DF4699, 1:200), Caveolin-1 (Proteintech, 16447-1-AP, 1:200), p21 (Santa cruz, sc-6246, 1:200), p65 (HUBIO, ET1603-12, 1:200), PTRF (Santa cruz, sc-51589, 1:200).

Following incubation and PBS rinses, the cardiomyocytes and deparaffinized heart sections were incubated with secondary antibody for 2 h and then the nuclei were stained with DAPI for 15 min. Imaging was performed using a confocal laser scanning microscope (Leica TCS SP8, Wetzlar, Germany). The total tissue positive areas were quantified using ImageJ software (version 1.8.0), with results presented as the percentage of positively stained area relative to the total area.

**RNA-sequencing**

In compliance with the manufacturer’s guidelines, TRIzol reagent (cat no.15596018, Invitrogen, Carlsbad, CA, USA) was employed for RNA extraction from heart tissue. The RNA integrity was assessed by electrophoresis with denaturing agarose gel. Then the poly(A) RNA was fragmented into small pieces and was reverse-transcribed to create the cDNA by SuperScript™ II Reverse Transcriptase (Invitrogen, cat. 1896649, USA). After a series of pretreatments, the ligated products were amplified with PCR. The average insert size for the final cDNA library was 300 ± 50 bp. Finally, 2 × 150 bp paired-end sequencing (PE150) was performed on an Illumina Novaseq™ 6000 (LC-Bio Technology Co., Ltd., Guangzhou, China).

**Statistical Analysis**

Statistical analysis of the experimental data was performed using GraphPad Prism 8.0.2 (Graph Pad Software, San Diego, CA). Student's t-test was employed for comparisons between two independent groups, while analysis of variance (ANOVA) with Tukey's multiple comparisons test was used for comparisons involving three or more groups. Values were considered statistically significant if P < 0.05. All biological and technical experiments were performed at least three times, and the data are presented as mean ± SEM, unless noted otherwise.

**Supplementary Figures**


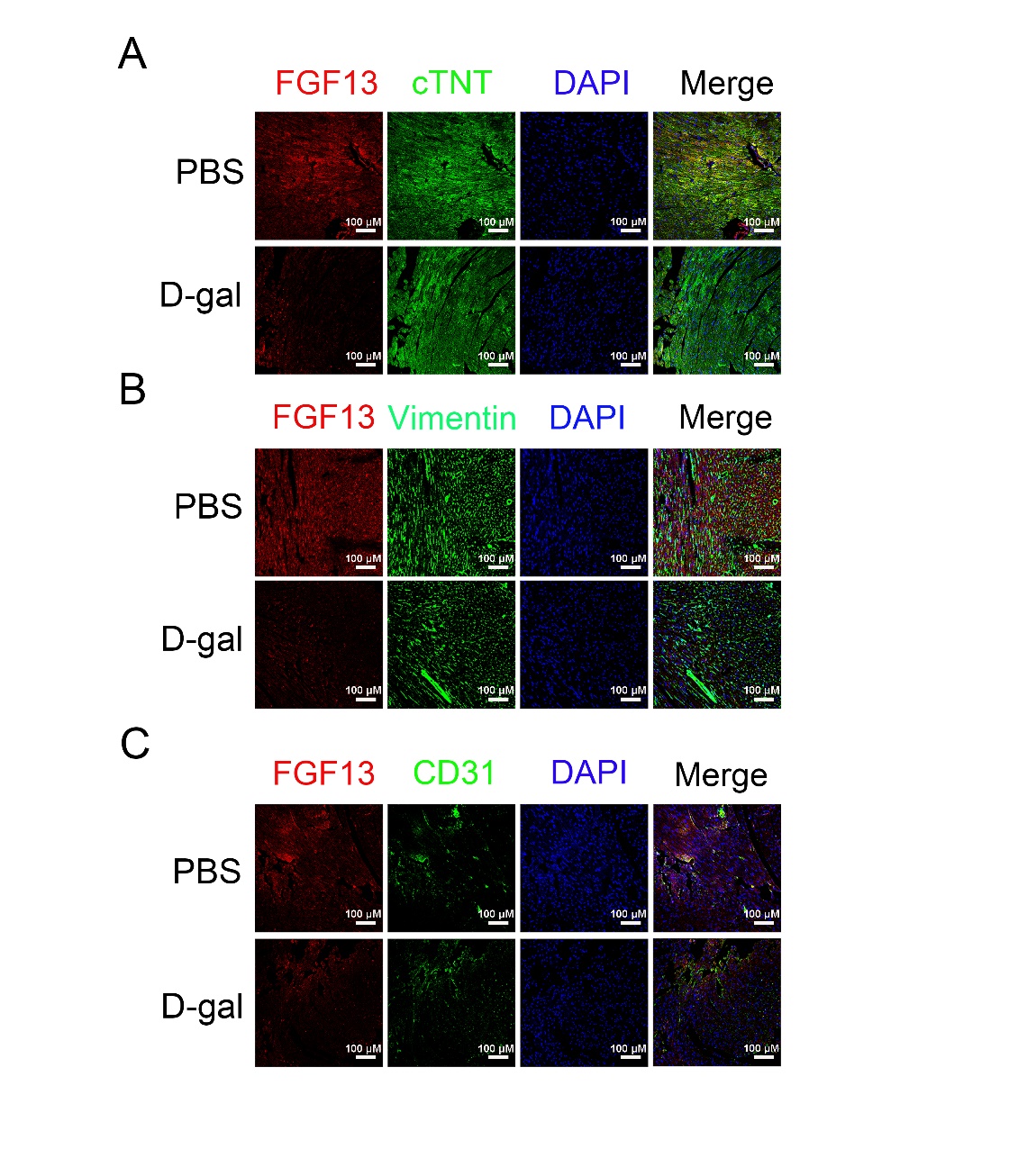


**Figure S1: The abundance of FGF13 is significantly downregulated in premature cardiac aging models induced by D-galactose.**

FGF13 is downregulated in cardiomyocytes upon D-galactose induced cardiac premature aging models. **(A–C)** Representative images of immunofluorescence staining of **(A)** FGF13 (red), c-TNT (green) and DAPI (blue), **(B)** FGF13 (red), vimentin (green) and DAPI (blue) and **(C)** FGF13 (red), CD31 (green) and DAPI (blue) in the mouse hearts in sham group and 8 weeks after D-galactose treatment. c-TNT: cardiomyocytes marker. Vimentin: fibroblasts marker. CD31: endothelial cells marker. Scale bar, 100 μm.


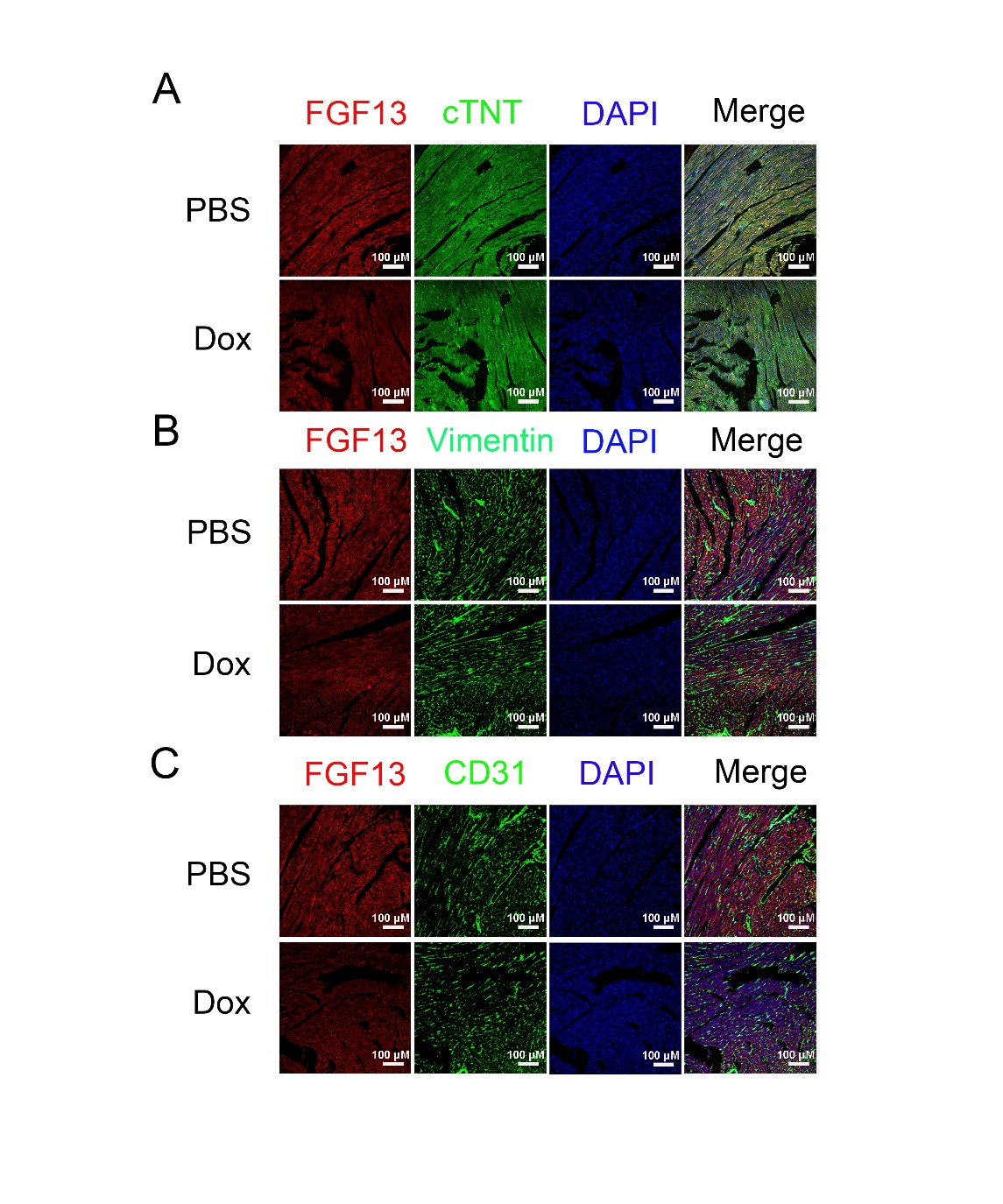


**Figure S2: the abundance of FGF13 is significantly downregulated in premature cardiac aging models induced by D-galactose.**

FGF13 is downregulated in cardiomyocytes upon Doxorubicin induced cardiac premature aging models. **(A–C)** Representative images of immunofluorescence staining of **(A)** FGF13 (red), c-TNT (green) and DAPI (blue), **(B)** FGF13 (red), Vimentin (green) and DAPI (blue) and **(C)** FGF13 (red), CD31 (green) and DAPI (blue) in the mouse hearts in sham group and 8 weeks after D-galactose treatment. c-TNT: cardiomyocytes marker. Vimentin: fibroblasts marker. CD31: endothelial cells marker. Scale bar, 100 μm.


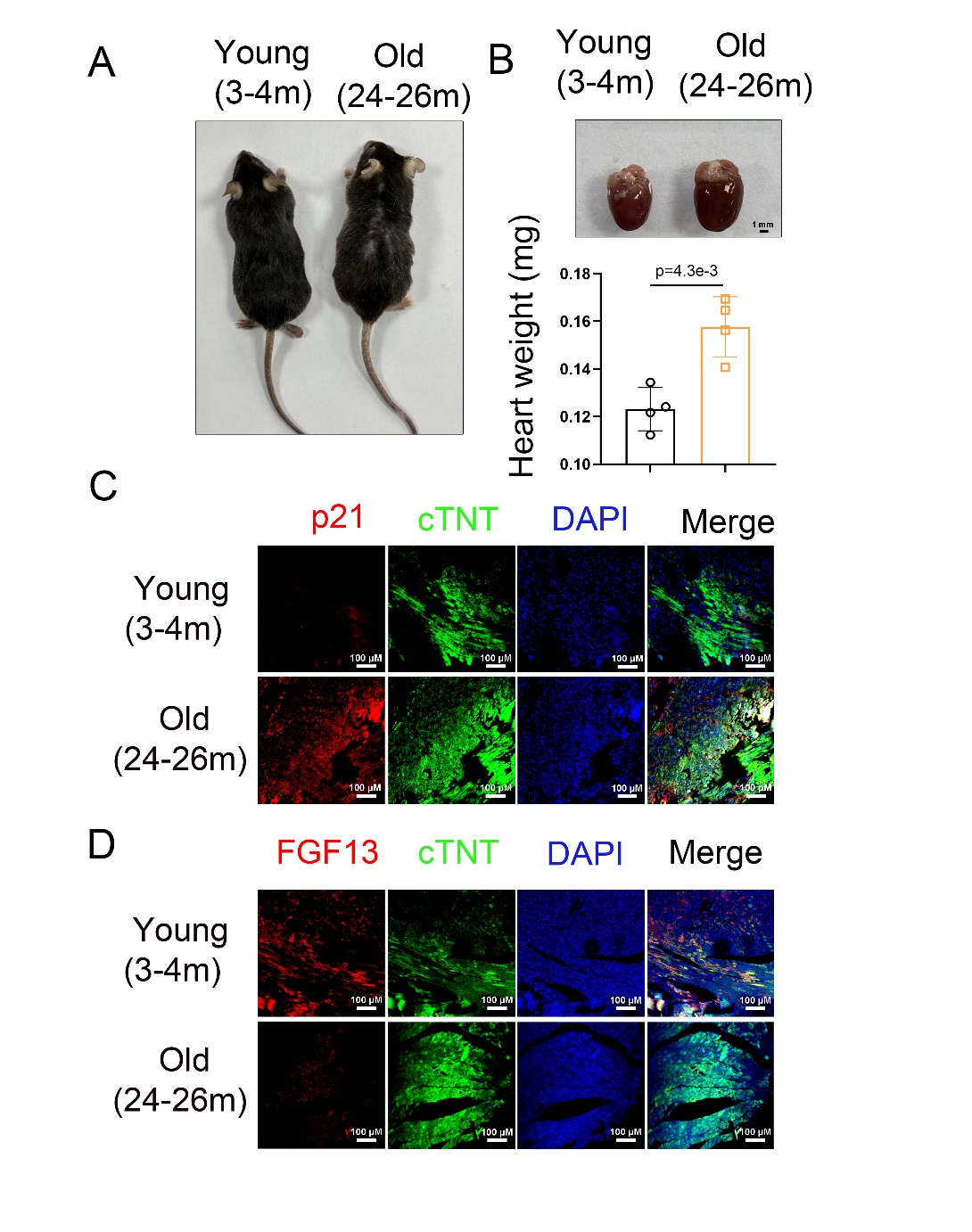


**Figure S3: The abundance of FGF13 is significantly downregulated in the heart of natrual aging mice.**

**(A-D)** 3-4 months young mice and 24-26 months aging mice were selected for subsequent studies. **(A)** Representative photograph of 3-4 young and 24-26 months aging mice (n=4 per group). **(B)** Representative whole heart images (scale bar, 1 mm) and representative data of HW in the indicated groups (n=4 per group). **(C-D)** Representative images of immunofluorescence staining of **(C)** p21 (red), c-TNT (green) and DAPI (blue), and **(D)** FGF13 (red), c-TNT (green) and DAPI (blue) in the mouse hearts in young group and aging group (n=4 per group). c-TNT: cardiomyocyte marker. Scale bar, 100 μm. Data are means ± SEM.  The P value was determined using two-tailed unpaired Student’s t test.


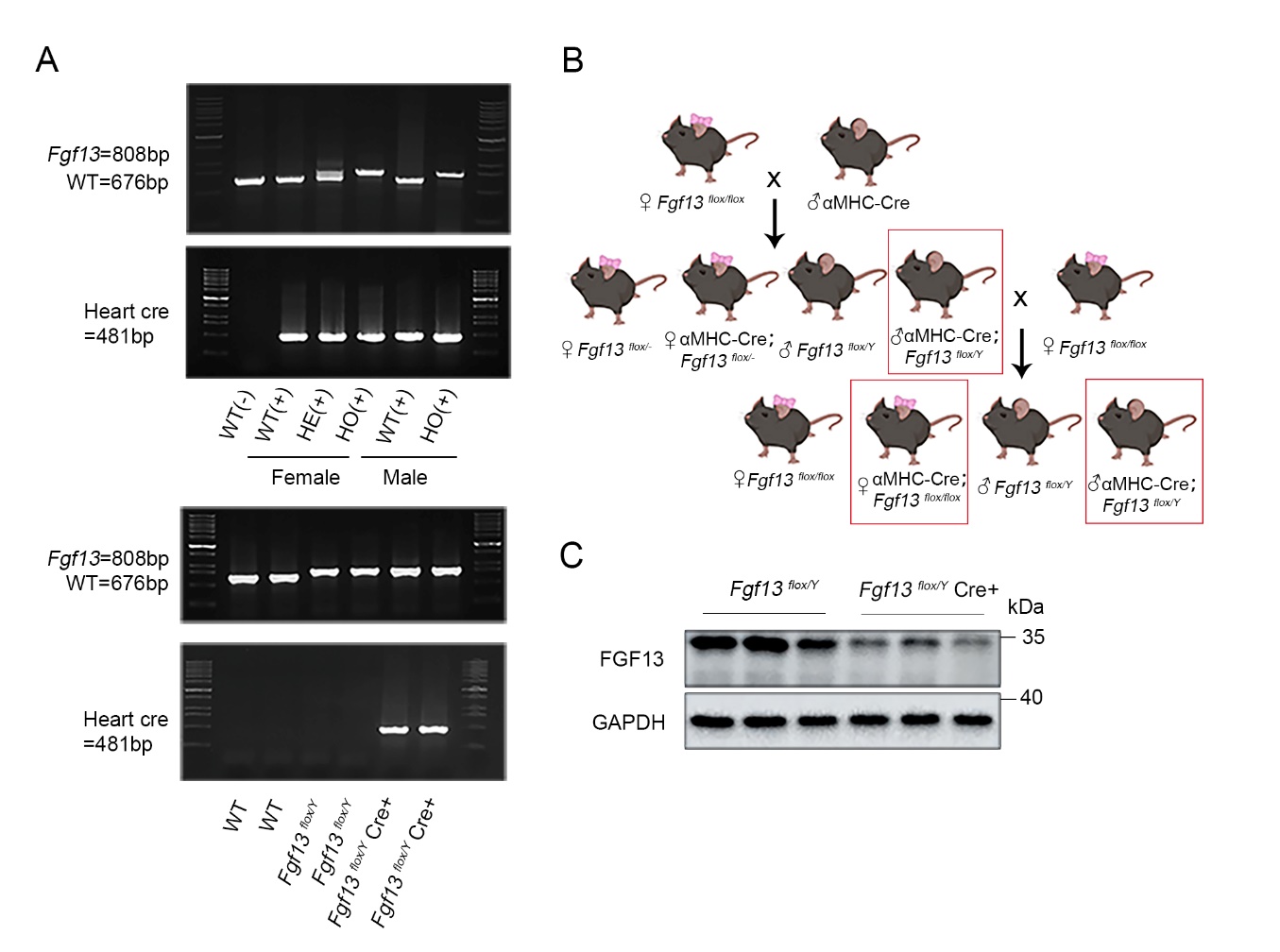


**Figure S4: Generation of cardiomyocyte-specific *Fgf13* knockout mice (αMHC-MerCreMer, *Fgf13^f/Y^ or Fgf13^f/f^*) and genotype identification.** For 6 or 7 weeks old wild-type (WT, *Fgf13^f/Y^ and Fgf13^f/f^* ) and *Fgf13*-KO (*Fgf13^f/Y^ and Fgf13^f/f^* crossed with αMHC-MerCreMer) mice, were intraperitoneally injected with tamoxifen at the dose of 75 mg/kg/day for 5 consecutive days, and kept for a 14-day waiting period to get the efficient gene knockout. **(A)** Genotyping was confirmed by tail preparation and sqRT-PCR at 2 weeks of age. **(B)** A diagram showing the strategy for the generation of *Fgf13*-KO male mice. **(C)** Immunoblotting for the expression of FGF13 in cardiac tissues in wild-type (WT, *Fgf13^f/Y^*) and *Fgf13*-KO (*Fgf13^f/Y^* crossed with αMHC-MerCreMer) mice injected with tamoxifen. (n=3 per group). −, Cre recombinase–negative mice; +, Cre recombinase–positive mice; bp, base pair; HE, heterozygous; HO, homozygous; WT, wild type.


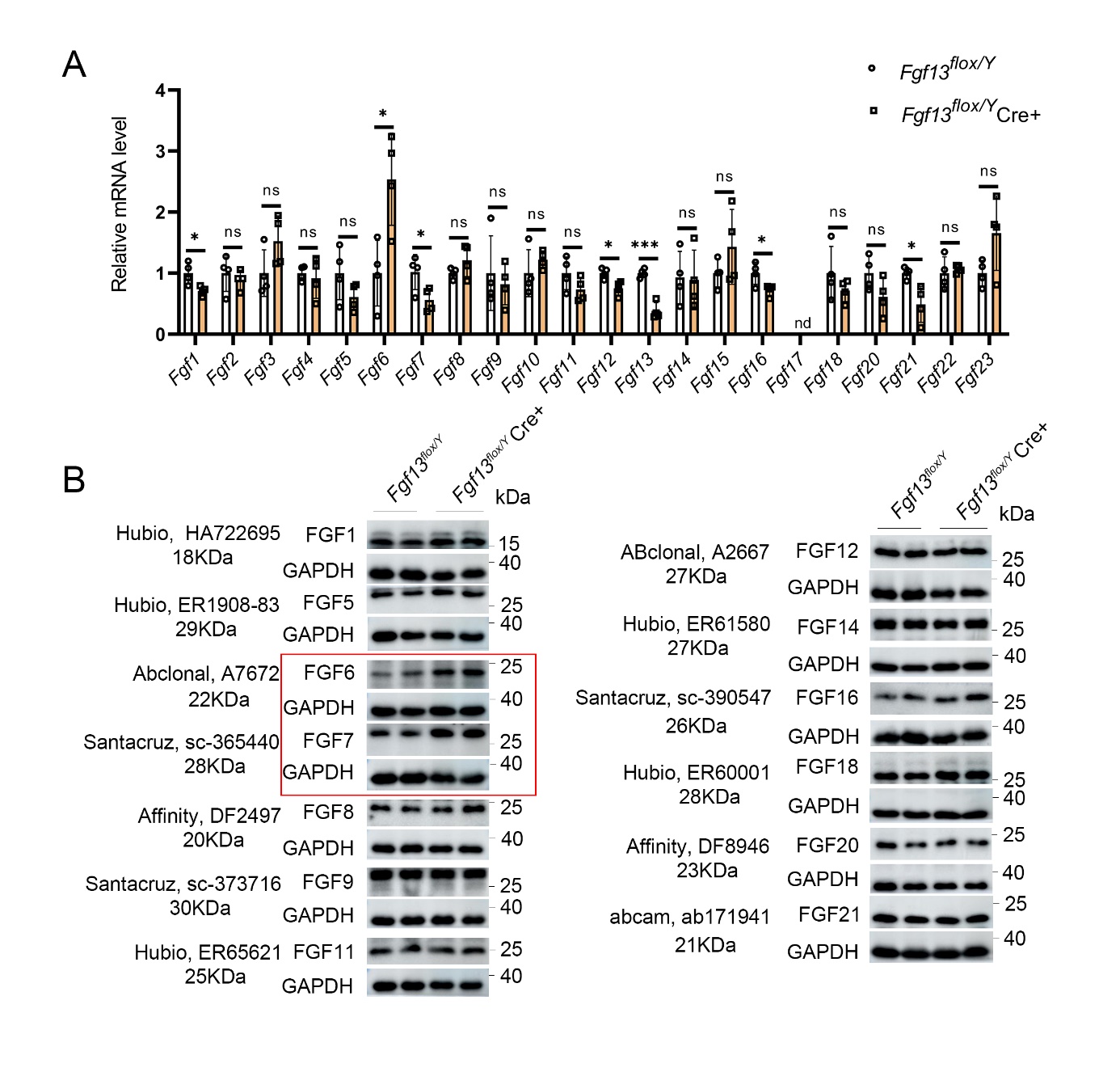


**Figure S5: Alterations in gene and protein expression levels of FGF family members in cardiac-specific Fgf13 knockout mice**

For 6 or 7 weeks old wild-type (WT, *Fgf13^f/Y^* ) and *Fgf13*-KO (*Fgf13^f/Y^* crossed with αMHC-MerCreMer) mice, were intraperitoneally injected with tamoxifen at the dose of 75 mg/kg/day for 5 consecutive days, and kept for a 14-day waiting period to get the efficient gene knockout. **(A)** Real-time qPCR analysis in mRNA levels of FGF family members in myocardium (n=4 per group). **(B)** Representative western blotting results in protein levels of FGF family members in myocardium. The protein level was standardized by GAPDH (n=4 per group). Data are means ± SEM. The P value was determined using two-tailed unpaired Student’s t test.


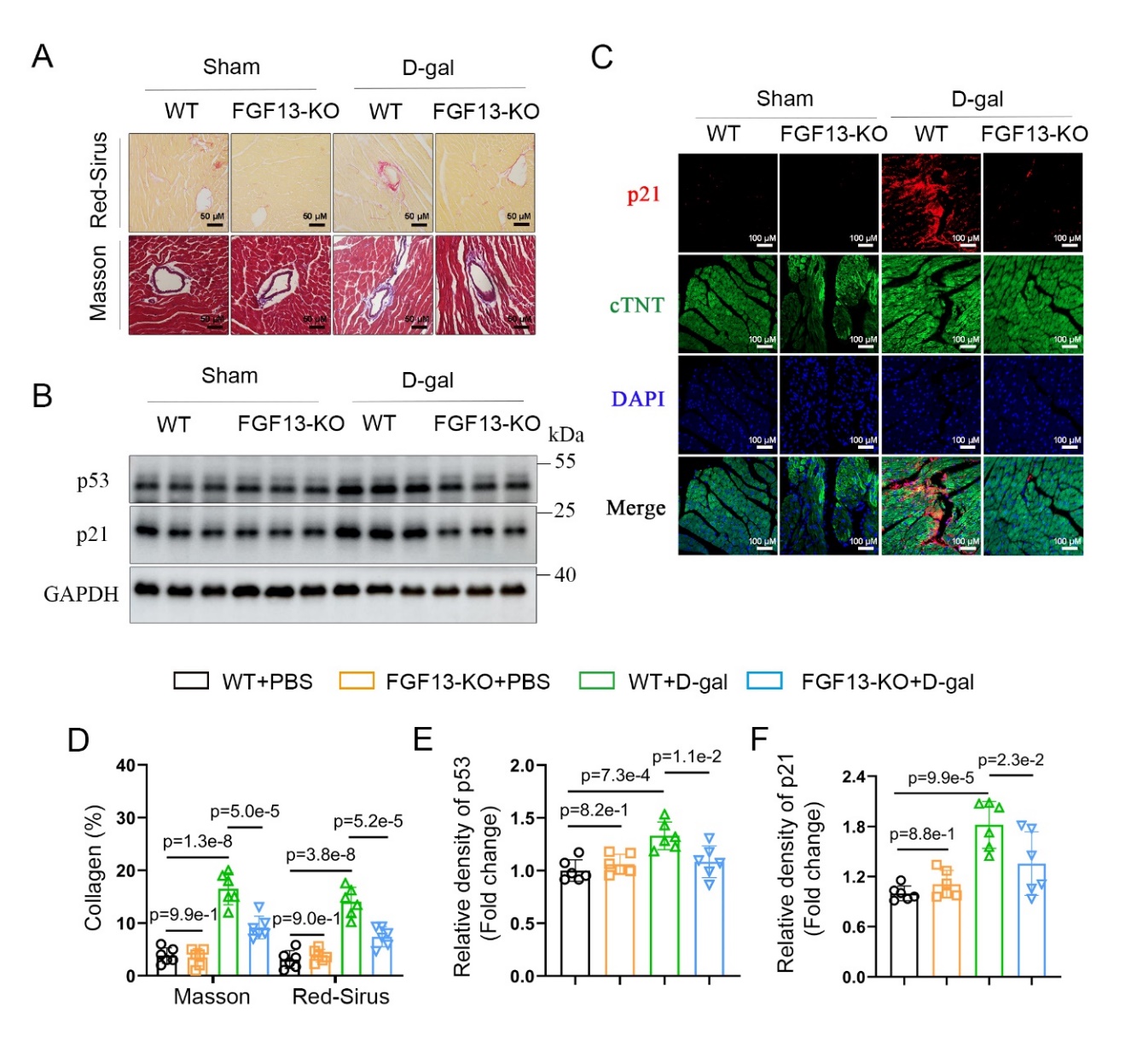


**Figure S6: Cardiac-specific knockout of *Fgf13* in cardiomyocytes alleviates D-galactose-induced cardiomyocyte senescence and cardiac injury.**

For 7 weeks old wild-type (WT, *Fgf13^f/Y^*) mice and *Fgf13KO* (*Fgf13^f/Y^* crossed with αMHC-MerCreMer) mice, tamoxifen was administered at the dose of 75 mg/kg/day for 5 consecutive days. One week after the injection, these mice were subjected to Sham or D-galactose treatment. **(A)** Masson staining (scale bar, 50 μm, left) and Sirius Red staining (scale bar, 50 μm, right) and **(D)** quantification (left) (n=6 per group). **(B)** Representative western blotting results and **(E-F)** related quantification of p53, p21. (n=6 per group). The protein level was standardized by GAPDH. **(C)** Representative images of immunofluorescence staining of p21 (red) and cTNT (green) and DAPI (blue) in D-galactose induced mouse hearts after myocardial specific *Fgf13* knockout (n=3 per group) (scale bar, 100 μm). Data are means ± SEM. The P value was determined using ANOVA with Tukey's multiple comparisons test.


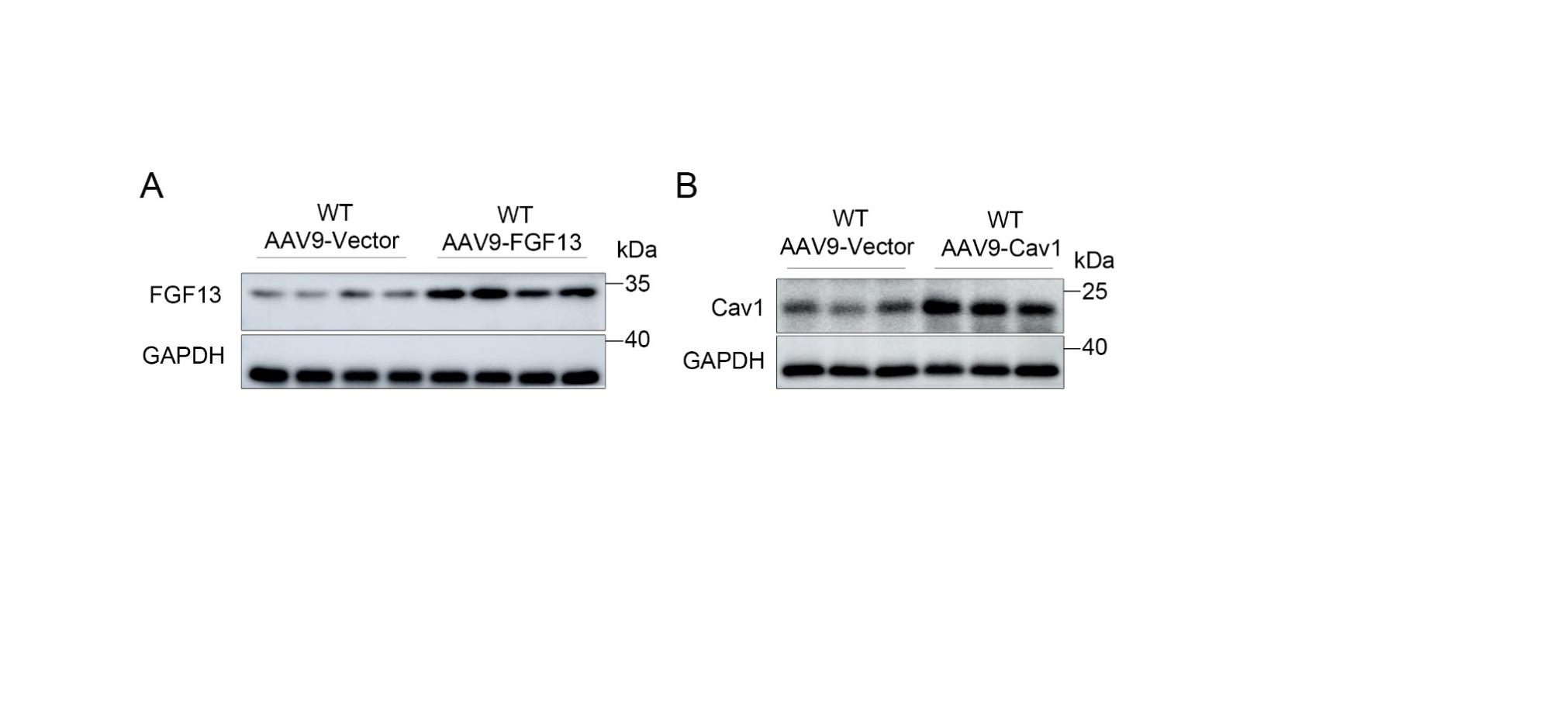


**Figure S7: Verification of FGF13 and Cav1 protein overexpression by AAV9 in mouse heart tissue.** **(A-B)** Representative western blotting for FGF13 **(A)** and Cav1 **(B)** expression in heart tissues. n=6. The protein level was standardized by GAPDH.

**
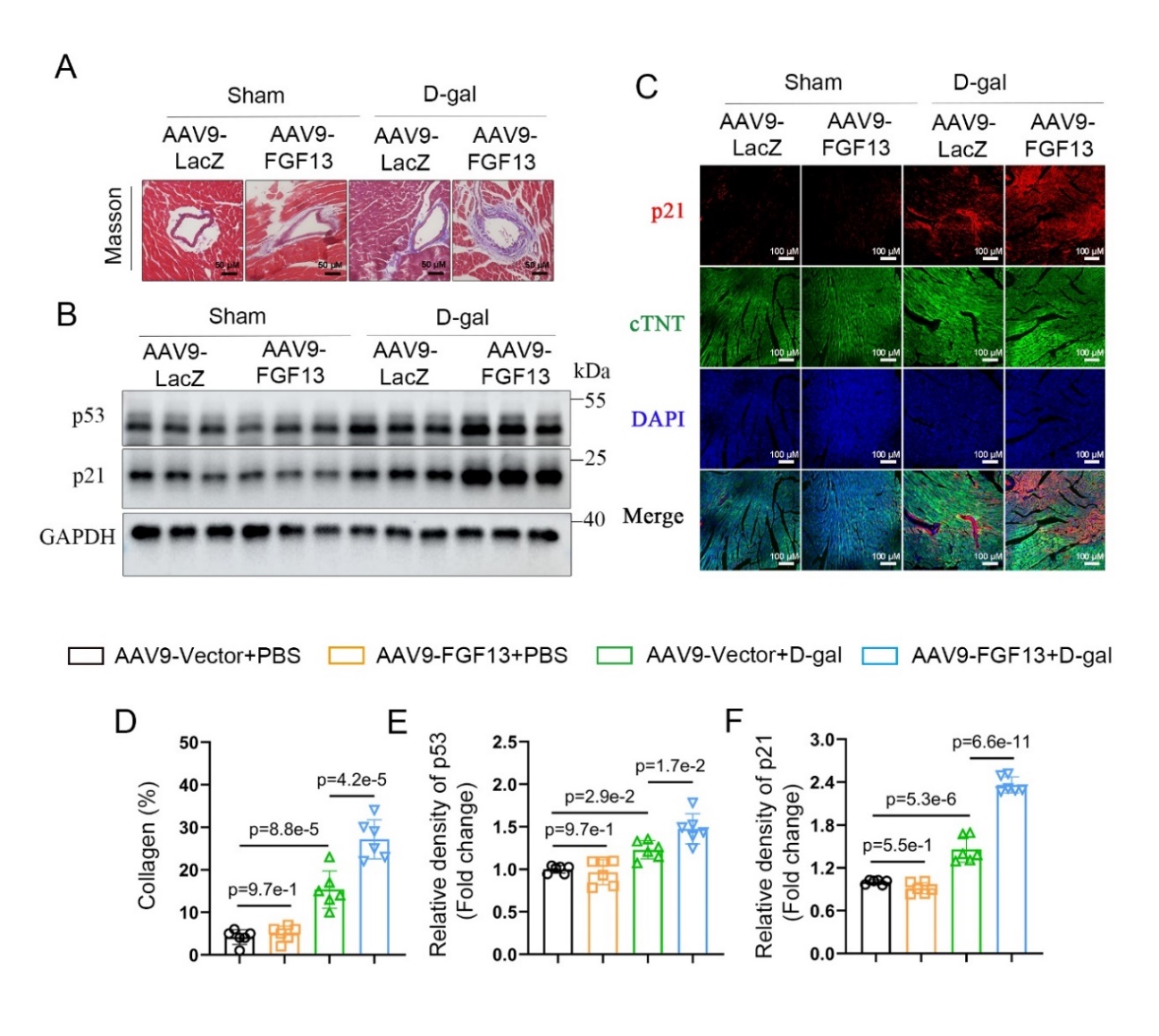
**

**Figure S8: Cardiac-specific FGF13 overexpression in cardiomyocytes upregulates D-galactose-induced cardiomyocyte senescence and cardiac injury.**

FGF13 overexpression vector (AAV9-cTnT-FGF13) and control vector (AAV9-LacZ) were injected intravenously into tail veins of 6 weeks old male C57BL/6J mice, respectively. 2 weeks after the injection, these mice were subjected to Sham or D-galactose treatment for 8 weeks. **(A)** Masson staining (scale bar, 50 μm) and **(D)** quantification (n=6 per group). **(B)** Representative western blotting results and **(E-F)** related quantification of p53, p21. (n=6 per group). The protein level was standardized by GAPDH. **(C)** Representative images of immunofluorescence staining of p21 (red) and cTNT (green) and DAPI (blue) in D-galactose induced mouse hearts after myocardial specific FGF13 overexpression (n=3 per group) (scale bar, 100 μm). Data are means ± SEM. The P value was determined using ANOVA with Tukey's multiple comparisons test.


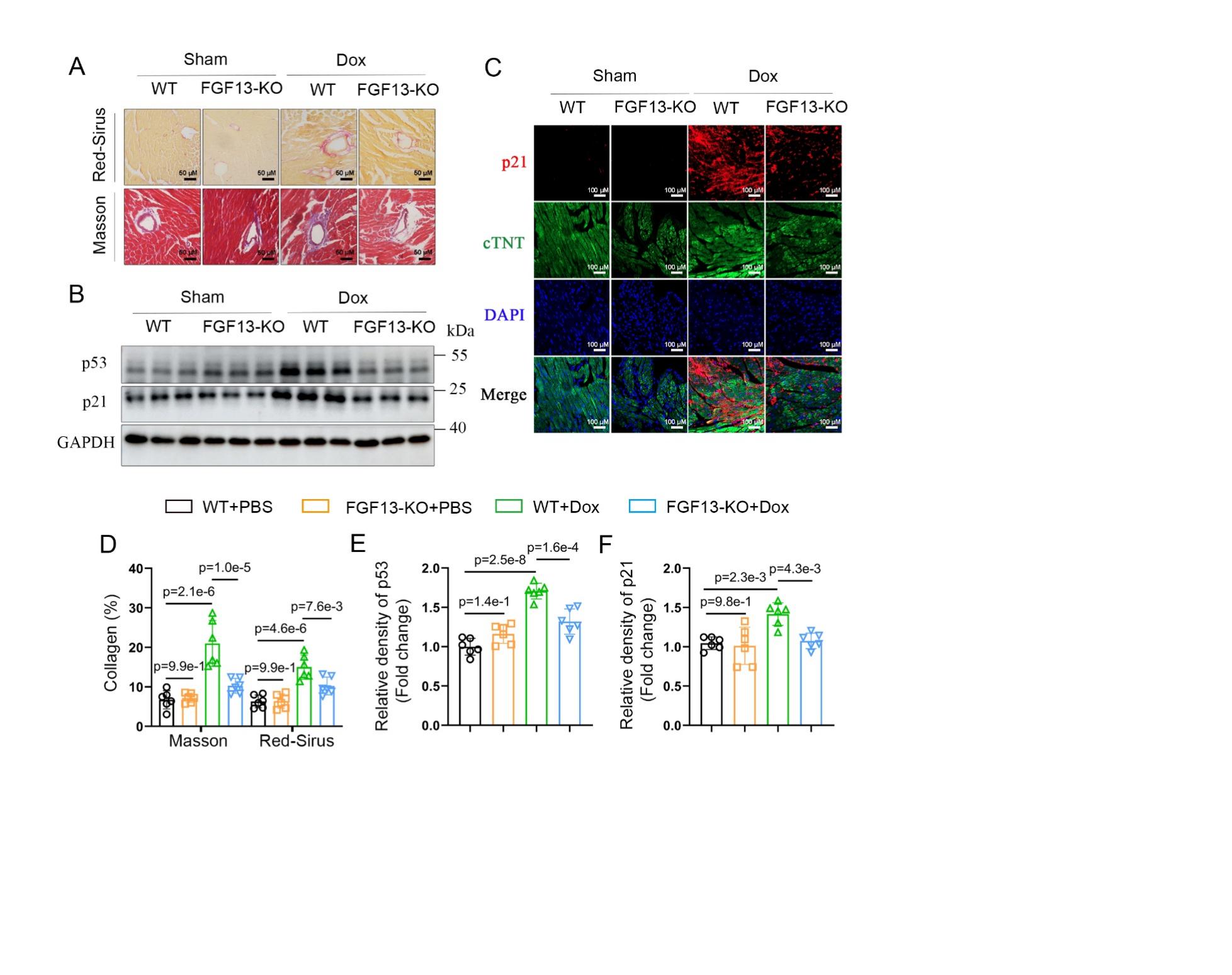


**Figure S9: Cardiac-specific knockout of *Fgf13* in cardiomyocytes alleviates Doxorubicin-induced cardiomyocyte senescence and cardiac injury.** For 7 weeks old wild-type (WT, *Fgf13^f/Y^*) mice and *Fgf13KO* (*Fgf13^f/Y^* crossed with αMHC-MerCreMer) mice, tamoxifen was administered at the dose of 75 mg/kg/day for 5 consecutive days. One week after the injection, these mice were subjected to Sham or Doxorubicin treatment. **(A)** Masson staining (scale bar, 50 μm, left) and Sirius Red staining (scale bar, 50 μm, right) and **(D)** quantification (left) (n=6 per group). **(B)** Representative western blotting results and **E, F** related quantification of p53, p21. (n=6 per group). The protein level was standardized by GAPDH. **(C)** Representative images of immunofluorescence staining of p21 (red) and cTNT (green) and DAPI (blue) in D-galactose induced mouse hearts after myocardial specific *Fgf13* knockout (n=3 per group) (scale bar, 100 μm). Data are means ± SEM. The P value was determined using ANOVA with Tukey's multiple comparisons test.

**
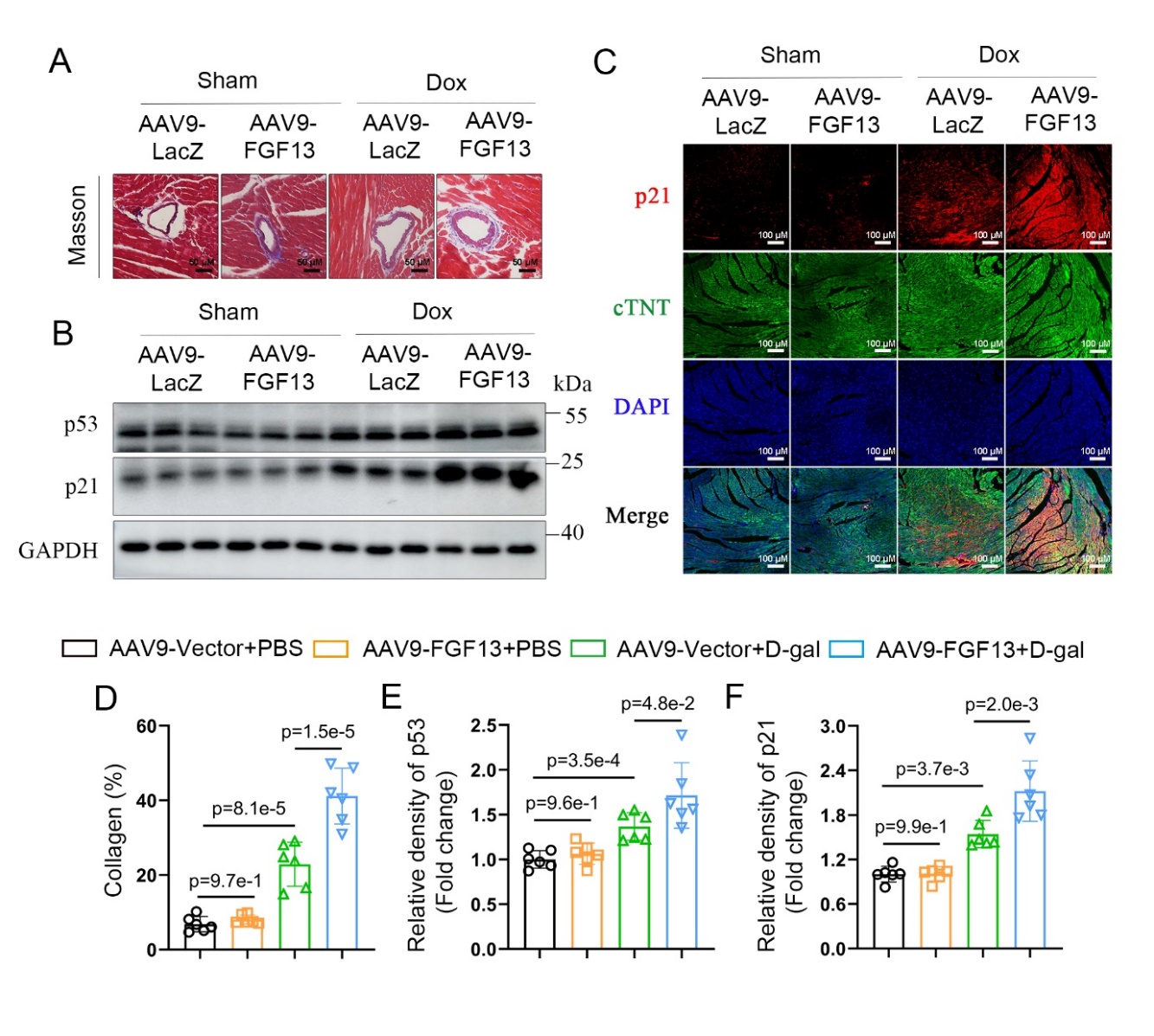
**

**Figure S10: Cardiac-specific FGF13 overexpression in cardiomyocytes upregulates Doxorubicin-induced cardiomyocyte senescence and cardiac injury.** FGF13 overexpression vector (AAV9-cTnT-FGF13) and control vector (AAV9-LacZ) were injected intravenously into tail veins of 6 weeks old male C57BL/ 6J mice, respectively. 2 weeks after the injection, these mice were subjected to Sham or Doxorubicin treatment for 8 weeks. **(A)** Masson staining (scale bar, 50 μm) and **(D)** quantification (left) (n=6 per group). **(B)** Representative Western blotting results and **(E-F)** related quantification of p53, p21. (n=6 per group). The protein level was standardized by GAPDH. **(C)** Representative images of immunofluorescence staining of p21 (red) and cTNT (green) and DAPI (blue) in Doxorubicin induced mouse hearts after myocardial specific FGF13 overexpression (n=3 per group) (scale bar, 100 μm). Data are means ± SEM. The P value was determined using ANOVA with Tukey's multiple comparisons test.

**
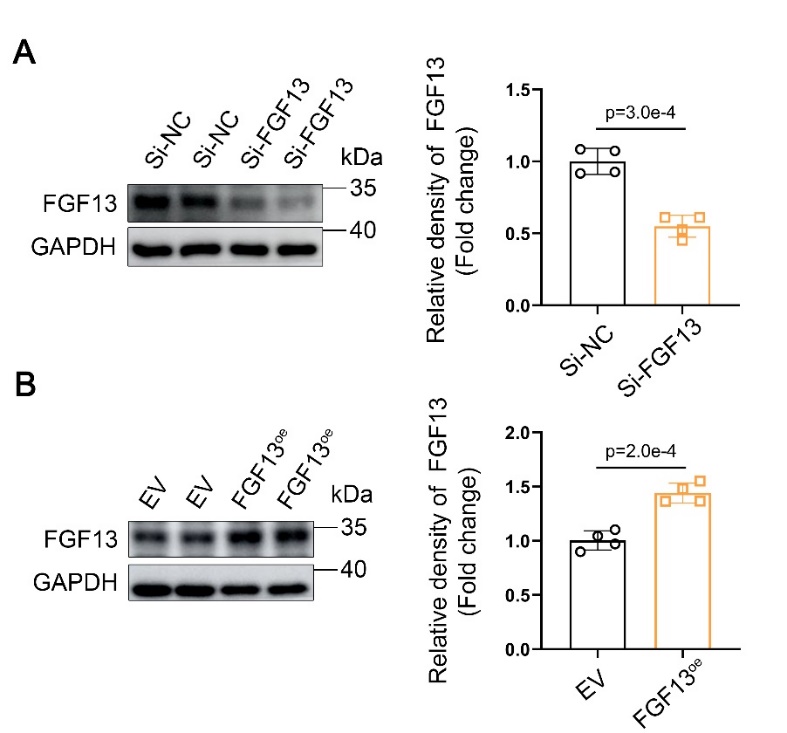
**

**Figure** **S11: Verification of the effects of FGF13 protein by a small interfering RNA for FGF13 and FGF13 overexpressing-plasmid in NRCMs. (A-B)** Representative western blotting for FGF13 expression in NRCMs transfected with **(A)** si-FGF13 (or si-NC) and **(B)** FGF13^oe^ (or EV), and densitometric quantification of FGF13 (n=4 per group). The protein level was standardized by GAPDH. Data are means ± SEM. The P value was determined using two-tailed unpaired Student’s t test.

**
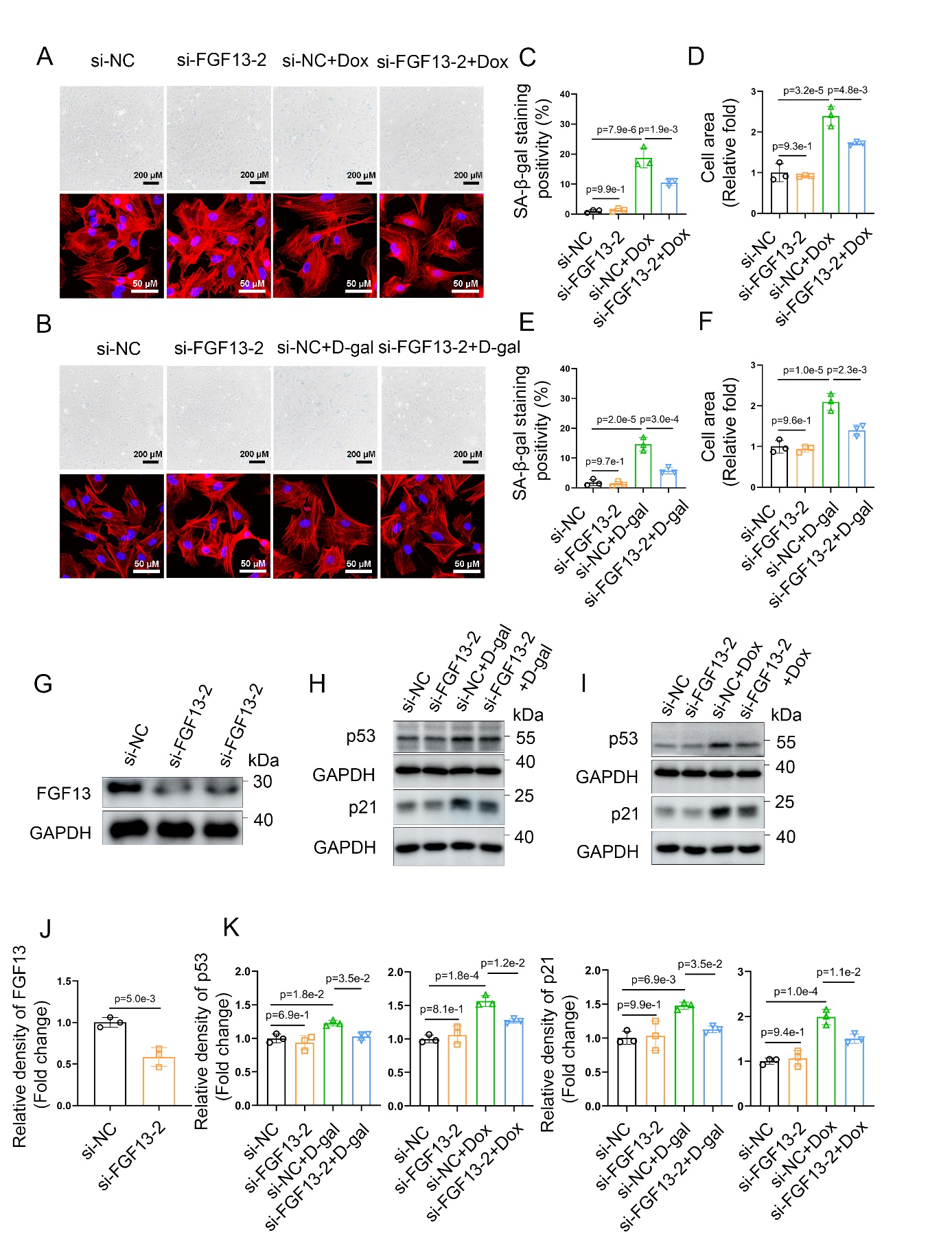
**

**Figure S12: FGF13 knockdown alleviates premature cardiomyocyte senescence using other independent siRNA sequences targeting FGF13.**

**(A-K)** NRCMs transfected with other independent siRNA sequences targeting FGF13 (si-FGF13-2) or si-NC, were either untreated or treated with Doxorubicin (0.1 μM) or D-galactose (20 g/L) for 72 h and related quantification. **(A-B)** *β-galactosidase* staining (the upper part) and **(C-D)** related quantification (n=3 per group). **(A-B)** TRITC Phalloidin staining (the lower part) and **(E-F)** related quantification (n=3 per group). **(G)** Representative western blotting for FGF13 expression in NRCMs transfected with si-FGF13-2 or si-NC, and **(J)** densitometric quantification of FGF13 (n=3 per group). **(H-I)** Representative western blotting for p53 and p21 in NRCMs and **(K)** related quantification (n=3 per group). The protein level was standardized by GAPDH. Data are means ± SEM. The P value was determined using two-tailed unpaired Student’s t test or ANOVA with Tukey's multiple comparisons test.

**
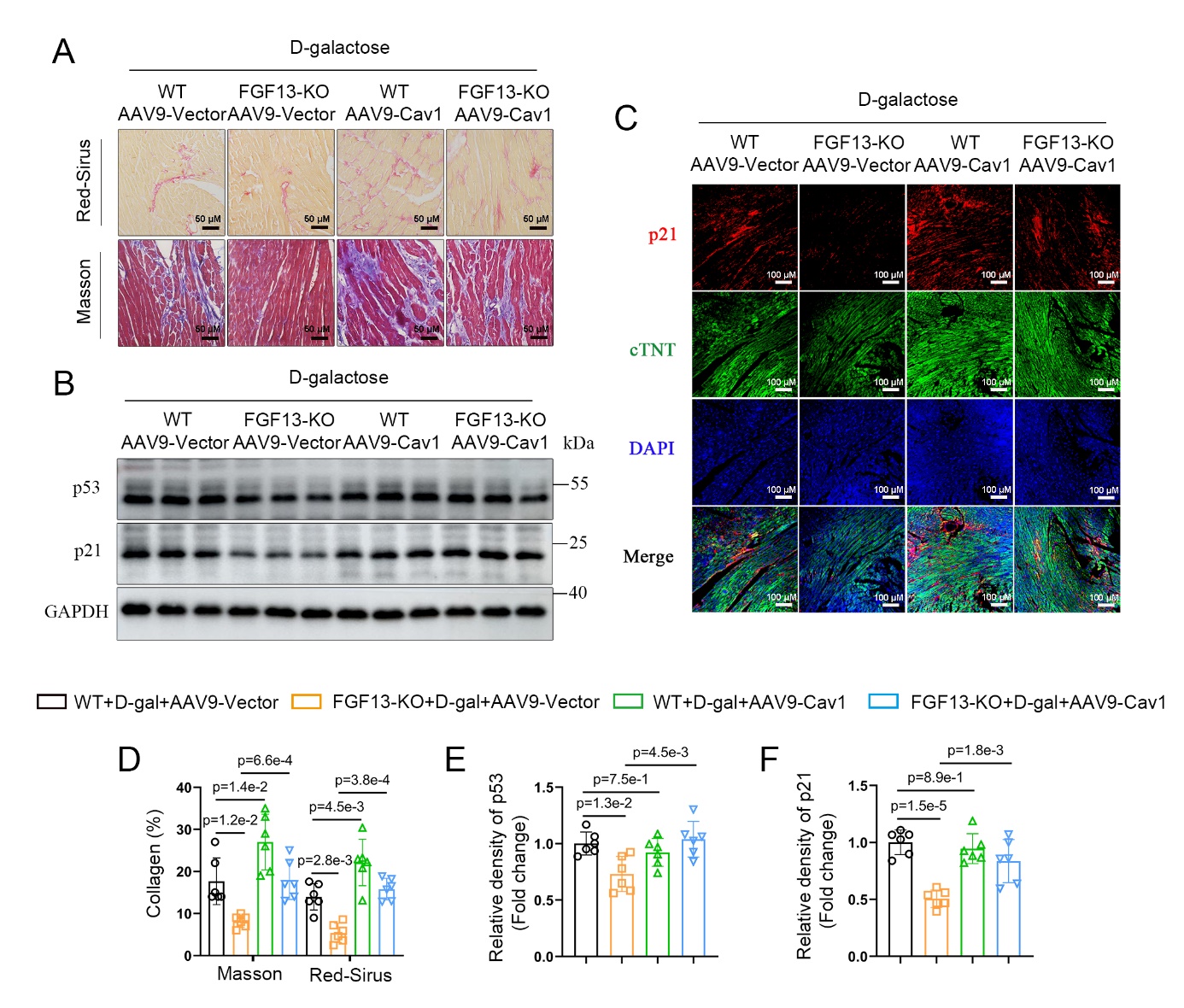
**

**Figure S13: Myocardial specific overexpression of Cav1 reverses the protective effect of *Fgf13* knockout against cardiomyocyte senescence and cardiac injury induced by D-galactose.** For 6 weeks old wild-type (WT, *Fgf13^f/Y^*) mice and *Fgf13KO* (*Fgf13^f/Y^* crossed with αMHC-MerCreMer) mice, tamoxifen was administered at the dose of 75 mg/kg/day for 5 consecutive days. On the 7th week, AAV9-Cav1 (or AAV9-LacZ) was injected into the tail vein for 2 weeks. Then these mice were subjected to D-galactose treatment. After successful modeling, cardiac function tests were performed, and tissue samples were collected. **(A)** Masson staining (scale bar, 50 μm, left) and Sirius Red staining (scale bar, 50 μm, right) and **(D)** quantification (left) (n=6 per group). **(B)** Representative Western blotting results and **(E-F)** related quantification of p53, p21. (n=6 per group). The protein level was standardized by GAPDH. **(C)** Representative images of immunofluorescence staining of p21 (red) and cTNT (green) and DAPI (blue) in mouse hearts (n=3 per group) (scale bar, 100 μm). Data are means ± SEM. The P value was determined using two-tailed unpaired Student’s t test or ANOVA with Tukey's multiple comparisons test.


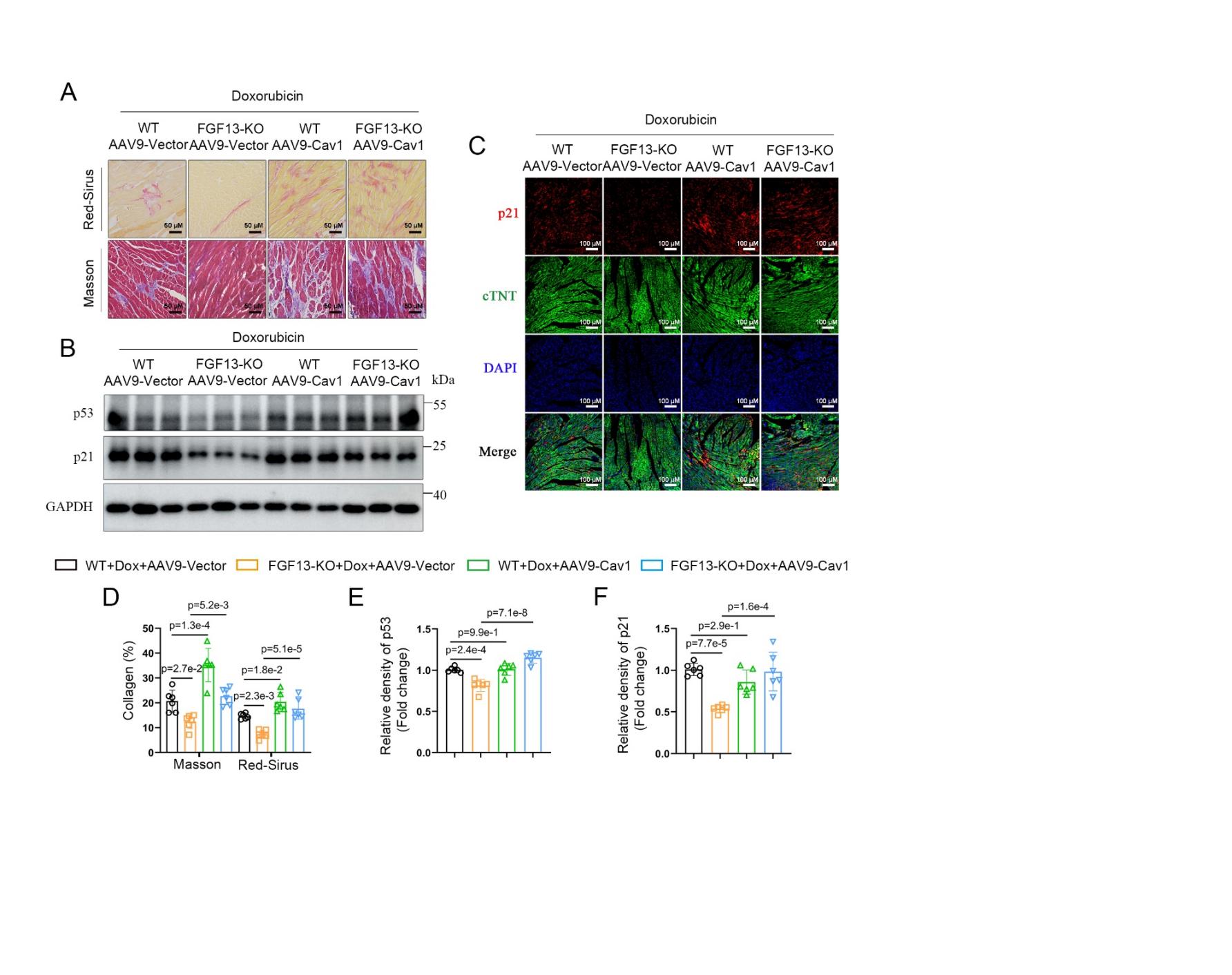


**Figure S14: Myocardial-specific overexpression of Cav1 reverses the protective effect of *Fgf13* knockout on cardiomyocyte senescence and cardiac injury induced by Doxorubicin.** For 6 weeks old wild-type (WT, *Fgf13^f/Y^*) mice and *Fgf13KO* (*Fgf13^f/Y^* crossed with αMHC-MerCreMer) mice, tamoxifen was administered at the dose of 75 mg/kg/day for 5 consecutive days. On the 7th week, AAV9-Cav1 (or AAV9-LacZ) was injected into the tail vein for 2 weeks. Then these mice were subjected to Doxorubicin treatment. After successful modeling, cardiac function tests were performed, and tissue samples were collected. **(A)** Masson staining (scale bar, 50 μm, left) and Sirius Red staining (scale bar, 50 μm, right) and **(D)** quantification (left) (n=6 per group). **(B)** Representative Western blotting results and **(E-F)** related quantification of p53, p21. (n=6 per group). The protein level was standardized by GAPDH. **(C)** Representative images of immunofluorescence staining of p21 (red) and cTNT (green) and DAPI (blue) in mouse hearts (n=3 per group) (scale bar, 100 μm). Data are means ± SEM. The P value was determined using two-tailed unpaired Student’s t test or ANOVA with Tukey's multiple comparisons test.

**
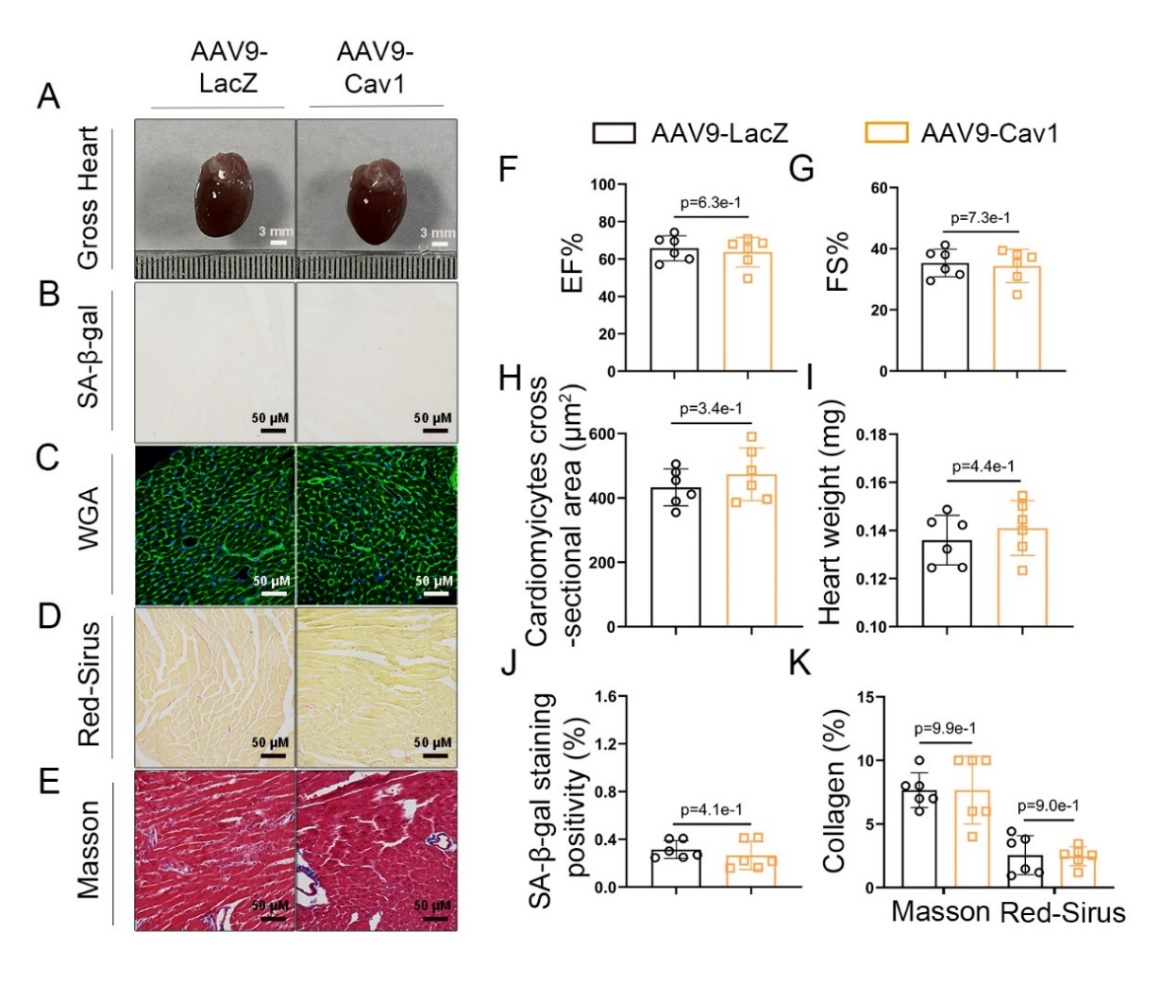
**

**Figure S15: Myocardial-specific overexpression of Cav1 is not sufficient to alter cardiac function and myocardial senescence.**

*Cav1* overexpression vector (AAV9-cTnT-Cav1) and control vector (AAV9-LacZ) were injected intravenously into tail veins of 6 weeks old male C57BL/6J mice, respectively. 8 Weeks after the injection, cardiac function tests were performed, and tissue samples were collected. **(A)** Representative whole heart images (scale bar, 3 mm). **(B)** Representative image of SA‐*β-gal* in the heart tissue (Arrows represent positive marks) (scale bar, 50 μm) and **(J)** quantification (n=6 per group). **(C)** WGA (wheat germ agglutinin; scale bar, 50 μm) and **(H)** quantification (n=6 per group). **(D)** Sirius Red staining (scale bar, 50 μm) and **(K)** quantification (left) (n=6 per group). **(E)** Masson staining (scale bar, 50 μm) and **(K)** quantification (right) (n=6 per group). **(F-G)** Representative echocardiographic data for LVEF and LVFS are shown (n=6 per group). **(I)** Representative data of heart weight in the indicated groups (n=6 per group). Data are means ± SEM. The P value was determined using two-tailed unpaired Student’s t test.


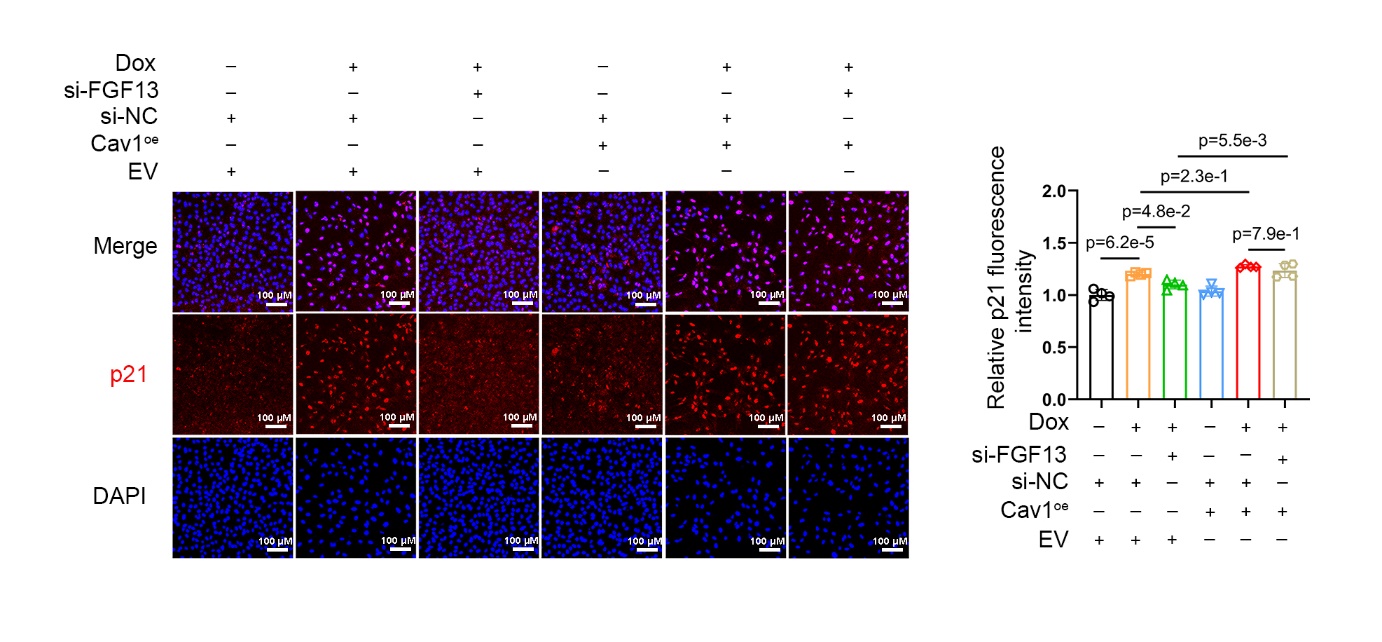


**Figure S16: The regulation of Doxorubicin-induced premature cardiomyocyte senescence by FGF13 in vitro depends on the expression of Cav1.**

Transfected NRCMs were either untreated or treated with Doxorubicin (0.1 μM) for 72 h. NRCMs were transfected with si-FGF13 (si-NC) and Cav1^oe^ (EV). Representative images of immunofluorescence staining of p21 (red) and DAPI (blue) (scale bar, 50 μm) (n=4 per group). Data are means ± SEM. The P value was determined using ANOVA with Tukey's multiple comparisons test.


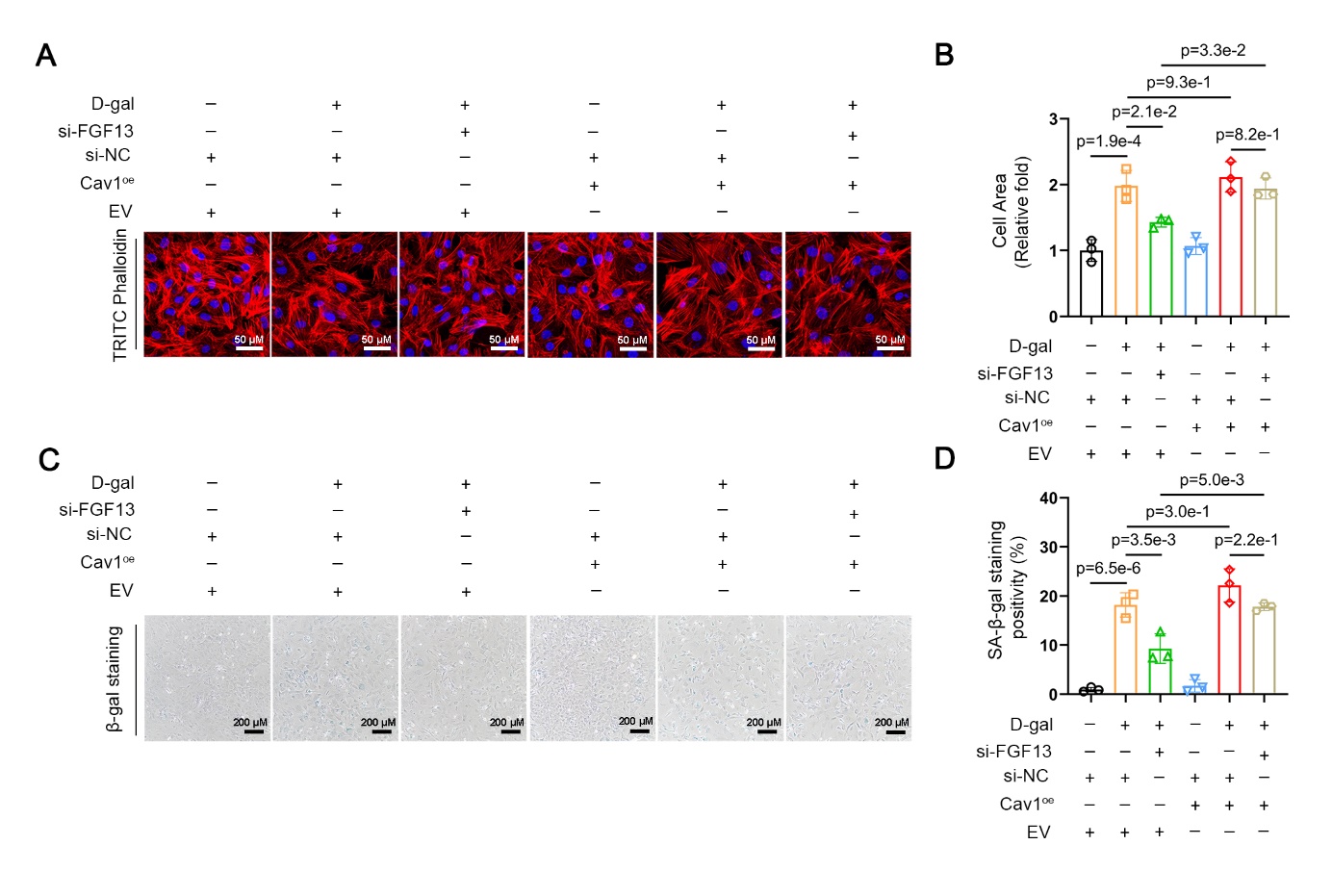


**Figure S17: The regulation of D-galactose-induced premature cardiomyocyte senescence by FGF13 in vitro depends on the expression of Cav1.**

Transfected NRCMs were either untreated or treated with D-galactose (20 g/L) for 72 h. **(A-D)** NRCMs were transfected with si-FGF13 (si-NC) and Cav1^oe^ (or empty vector). **(E)** TRITC Phalloidin staining in NRCMs, and **(B)** related quantification (n=4 per group). **(C)** *β-galactosidase* staining in NRCMs, and **(D)** related quantification (n=4 per group). Data are means ± SEM. The P value was determined using ANOVA with Tukey's multiple comparisons test.

**
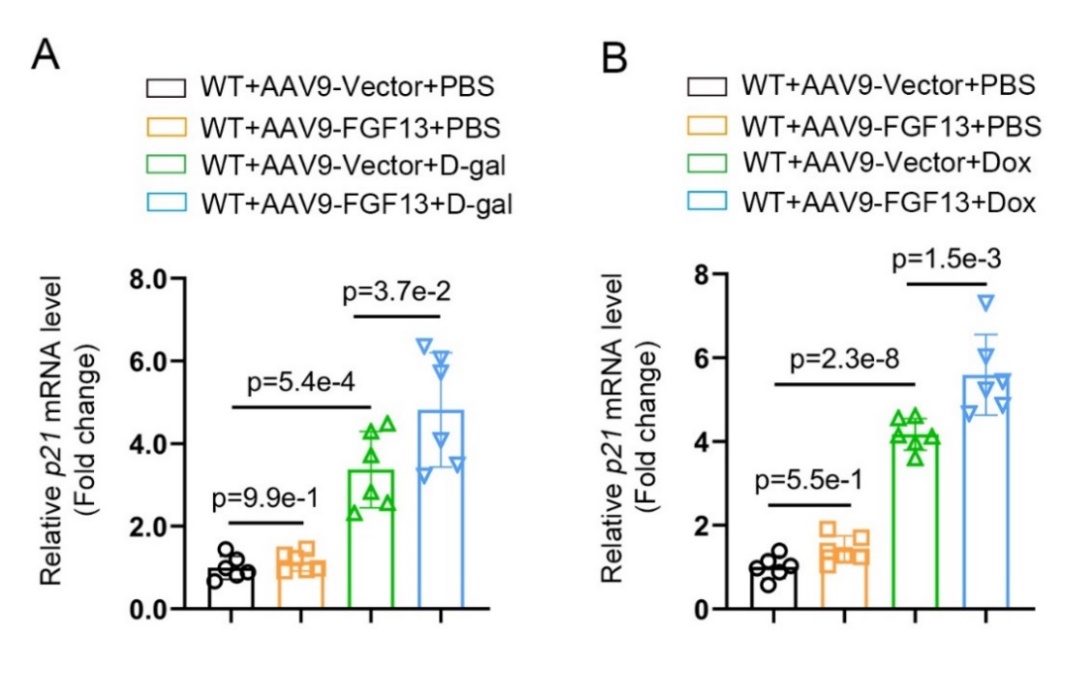
**

**Figure S18:** **Cardiac-specific FGF13 overexpression in cardiomyocytes upregulates premature cardiac aging.**

**(A-B)** Real-time qPCR analysis of *p21* in **(A)** D-galactose or **(B)** Doxorubicin induced mouse hearts after myocardial specific FGF13 overexpression (n=6 per group). Data are means ± SEM. The P value was determined using ANOVA with Tukey's multiple comparisons test.

**Supplementary Tables**

**Table S1**

| Gene | Primer Sequence |
| --- | --- |
| *p21* mouse F | 5'-CCTGGTGATGTCCGACCTG-3′ |
| *p21* mouse R | 5'-CCATGAGCGCATCGCAATC-3′ |
| *Gapdh* mouse F | 5'-ACTTGAAGGGTGGAGCCAAA-3' |
| *Gapdh* mouse R | 5'-GACTGTGGTCATGAGCCCTT-3’ |
| *Cav1* Rat F | 5'-CTACAAGCCCAACAACAAGGC-3' |
| *Cav1* Rat R | 5'-AGGAAGCTCTTGATGCACGGT-3' |
| *Cav2* Rat F | 5'-GCTCAACTCTCATCTCAAGCT-3' |
| *Cav2* Rat R | 5'-TCTGTCACACTCTTCCATATT-3' |
| *Cav3* Rat F | 5'-GGTGAACAGAGACCCCAAGA-3' |
| *Cav3* Rat R | 5'-GGAGACGGTGAAAGTGGTGT-3' |
| *Gapdh* Rat F | 5'-GAAGGGTGGGGCCAAAAG-3' |
| *Gapdh* Rat R | 5'-GGATGCAGGGATGATGTTCT-3' |

**Table S2**

| Primer | Sequence (5’→3’) | | | | Primer Type |
| --- | --- | --- | --- | --- | --- |
| P1 | AGCGCTCTAAAATTCAGGATGC | | | | Forward |
| P2 | CTTCAGGAATAAATGGGGACAGAT | | | | Reverse |
| PCR Reaction System | Reaction Component | | | | Volume (µL) |
|  | ddH_2_O | | | | 7.0 |
|  | 2×transTaq-T PCR SuperMix | | | | 10.0 |
|  | P1 (10 pmol/µL) | | | | 0.5 |
|  | P2 (10 pmol/µL) | | | | 0.5 |
|  | Genomic DNA | | | | 2 |
|  | Total | | | | 20 |
|  | 2×transTaq-T PCR SuperMix from TransGen Biotech  (Code number:AS122 ) | | | | |
| Cycling Reaction | Step | Temp | Time | Note | |
|  | 1 | 94℃ | 3 min |  | |
|  | 2 | 94℃ | 30 sec |  |  |
|  | 3 | 60℃ | 30 sec |  |  |
|  | 4 | 72℃ | 1 min | 35 repeats to 2 | |
|  | 5 | 72℃ | 5 min |  | |
|  | 6 | 12℃ | Hold |  |  |
| Genotype | Mutant =808 bp Wild type =676 bp | | | | |

**Table S3.**

The target sequences for siRNAs.

| **siRNA** |  | **Sequence** |
| --- | --- | --- |
| si-NC | Forward | UUCUCCGAACGUGUCACGUTT |
|  | Reverse | ACGUGACACGUUCGGAGAATT |
| si-FGF13 | Forward | GCACUUACACUCUGUUUAACCTT |
|  | Reverse | GGUUAAACAGAGUGUAAGUGCTT |
| si-FGF13-2 | Forward | GCGUCUCUGGUGUACUGAAUGTT |
|  | Reverse | CAUUCAGUACACCAGAGACGCTT |
| si-Cav1 | Forward | GGCAAGAUAUUCAGCAAUAUCTT |
|  | Reverse | GAUAUUGCUGAAUAUCUUGCCTT |

**Table S4**

Echocardiographic parameters in D-galactose-induced mouse experiment.

|  | PBS+  WT | PBS+  FGF13-KO | D-galactose+  WT | D-galactose+  FGF13-KO |
| --- | --- | --- | --- | --- |
| LVFS (%) | 41.52±8.53 | 37.15±8.29 ^ns^ | 26.14±4.20** | 35.48±5.44^#^ |
| LVEF (%) | 72.90±6.82 | 67.67±6.01 ^ns^ | 52.37±7.20*** | 66.09±7.25^#^ |
| BW(g) | 30.15±1.68 | 29.73±1.83 ^ns^ | 27.83±1.48 ^ns^ | 29.49±1.96 ^NS^ |
| HW (mg) | 133±7 | 134±5 ^ns^ | 156±7**** | 142±6^##^ |
| HW/BW (mg/g) | 4.43±0.28 | 4.53±0.34 ^ns^ | 5.60±0.28**** | 4.84±0.34^##^ |

ns, represents p >0.05 vs WT + PBS group, *, p <0.05, **, p <0.01, ***, p <0.001, ****, p <0.0001. NS, represents p >0.05 vs WT + D-galactose group, #, p <0.05, ##, p <0.01, ###, p < 0.001, ####, p <0.0001. LVEF, LV ejection fraction; LVFS, LV fractional shortening; HW, Height Weight; HW/BW, the ratio of height weight to body weight. All measurements are means ± SD. Data were analyzed by ANOVA with Tukey's multiple comparisons test. n.s. = not significant.

**Table S5**

Echocardiographic parameters in D-galactose-induced mouse with treatment of AAV9 (Empty Vector and FGF13)

|  | PBS+  AAV9-LacZ | PBS+  AAV9-FGF13 | D-galactose+  AAV9-LacZ | D-galactose+  AAV9-FGF13 |
| --- | --- | --- | --- | --- |
| LVFS (%) | 39.18±8.88 | 42.74±4.70 ^ns^ | 26.63±3.77** | 18.79±5.25^#^ |
| LVEF (%) | 69.93±10.19 | 74.85±6.93 ^ns^ | 53.20±4.59*** | 39.22±9.52^##^ |
| BW(g) | 30.41±2.05 | 30.11±2.14 ^ns^ | 28.43±1.65 ^ns^ | 27.82±0.87 ^NS^ |
| HW (mg) | 135±6 | 137±5 ^ns^ | 163±8**** | 181±4^###^ |
| HW/BW (mg/g) | 4.45±0.26 | 4.55±0.28 ^ns^ | 5.75±0.22**** | 6.51±0.19^####^ |

ns, represents p > 0.05 vs PBS+ AAV9-LacZ group, *, p < 0.05, **, p < 0.01; NS, represents p >0.05 vs AAV9-LacZ + D-galactose group, #, p < 0.05, ##, p < 0.01, ###, p < 0.001; LVEF, LV ejection fraction; LVFS, LV fractional shortening; HW, Height Weight; HW/BW, the ratio of height weight to body weight. All measurements are means ± SD. Data were analyzed by ANOVA with Tukey's multiple comparisons test. n.s. = not significant.

**Table S6**

Echocardiographic parameters in Doxorubicin-induced mouse experiment.

|  | PBS+  WT | PBS+  FGF13-KO | Doxorubicin +  WT | Doxorubicin +  FGF13-KO |
| --- | --- | --- | --- | --- |
| LVFS (%) | 35.35±5.72 | 35.27± 11.57 ^ns^ | 22.42± 7.41** | 31.78± 7.45^#^ |
| LVEF (%) | 65.75±8.72 | 64.85± 14.41 ^ns^ | 45.58± 13.65** | 60.72± 11.76^#^ |
| BW(g) | 27.61±1.30 | 28.03±2.17 ^ns^ | 22.79±1.16**** | 22.64±0.81 ^NS^ |
| HW (mg) | 137±10 | 140±11 ^ns^ | 111±3**** | 126±5^#^ |
| HW/BW (mg/g) | 4.96±0.20 | 4.99±0.28 ^ns^ | 4.86±0.34 ^NS^ | 5.58±0.28^##^ |

ns, represents p >0.05 vs WT + PBS group, *, p <0.05, **, p <0.01, ***, p <0.001, ****, p <0.0001. NS, represents p >0.05 vs WT + Doxorubicin group, #, p <0.05, ##, p <0.01, ###, p < 0.001, ####, p <0.0001. LVEF, LV ejection fraction; LVFS, LV fractional shortening; HW, Height Weight; HW/BW, the ratio of height weight to body weight. All measurements are means ± SD. Data were analyzed by ANOVA with Tukey's multiple comparisons test. n.s. = not significant.

**Table S7**

Echocardiographic parameters in Doxorubicin-induced mouse with treatment of AAV9 (Empty Vector and FGF13)

|  | PBS+  AAV9-LacZ | PBS+  AAV9-FGF13 | Doxorubicin+  AAV9-LacZ | Doxorubicin +  AAV9-FGF13 |
| --- | --- | --- | --- | --- |
| LVFS (%) | 39.18±8.88 | 42.74±4.70 ^ns^ | 26.63±3.77* | 18.79±5.25^##^ |
| LVEF (%) | 67.97±6.54 | 66.92±8.43 ^ns^ | 53.11±9.77* | 39.10±12.61^##^ |
| BW(g) | 28.22±1.48 | 28.35±2.09 ^ns^ | 21.51±2.05**** | 21.04±1.35 ^NS^ |
| HW (mg) | 139±5 | 138±8 ^ns^ | 107±11**** | 90±8^##^ |
| HW/BW (mg/g) | 4.92±0.28 | 4.87±0.33 ^ns^ | 4.79±0.30 ^NS^ | 4.25±0.36^##^ |

ns, represents p >0.05 vs PBS+ AAV9-LacZ group, *, p <0.05, **, p <0.01; NS, represents p >0.05 vs AAV9-LacZ + Doxorubicin group, #, p <0.05, ##, p <0.01, ###, p <0.001; LVEF, LV ejection fraction; LVFS, LV fractional shortening; HW, Height Weight; HW/BW, the ratio of height weight to body weight. All measurements are means ± SD. Data were analyzed by ANOVA with Tukey's multiple comparisons test. n.s. = not significant.

**Table S8**

Echocardiographic parameters in D-galactose-induced mouse with treatment of AAV9 (Empty Vector, and Cav1)

|  | D-galactose+  WT+ AAV9- LacZ | D-galactose+  FGF13-KO+ AAV9-LacZ | D-galactose+  WT+  AAV9-Cav1 | D-galactose+  FGF13-KO+ AAV9-Cav1 |
| --- | --- | --- | --- | --- |
| LVFS (%) | 25.78±3.80 | 33.518±4.88*** | 20.60±4.30 ^##^ | 23.69±1.43^&&&&^ |
| LVEF (%) | 51.80±6.42 | 63.27±7.13*** | 42.52±7.54 ^# #^ | 48.66±2.72^&&&&^ |
| BW(g) | 28.30±1.23 | 29.28±1.04^ns-1^ | 29.15±1.41 ^ns-2^ | 29.42±1.16 ^ns-3^ |
| HW (mg) | 160±9 | 139±5* | 165±16 ^ns-2^ | 159±11^&^ |
| HW/BW (mg/g) | 5.65±0.24 | 4.74±0.20*** | 5.67±0.45 ^ns-2^ | 5.42±0.41^&^ |

ns-1, represents p >0.05 vs D-galactose+WT+ AAV9-LacZ group, *, p <0.05, **, p <0.01; ns-2, represents p >0.05 vs D-galactose+WT+ AAV9-LacZ group, ###, p <0.001, ####, p <0.0001; ns-3, represents p >0.05 vs D-galactose+FGF13-CKO+ AAV9-LacZ group, ^&^, p <0.05, ^&&^, p <0.01; LVEF, LV ejection fraction; LVFS, LV fractional shortening; HW, Height Weight; HW/BW, the ratio of height weight to body weight. All measurements are means ± SD. Data were analyzed by ANOVA with Tukey's multiple comparisons test. n.s. = not significant.

**Table S9**

Echocardiographic parameters in Doxorubicin-induced mouse with treatment of AAV9 (Empty Vector, and Cav1)

|  | Doxorubicin+  WT+  AAV9-LacZ | Doxorubicin +  FGF13-KO+ AAV9-LacZ | Doxorubicin+  WT+  AAV9-Cav1 | Doxorubicin+  FGF13-KO+ AAV9-Cav1 |
| --- | --- | --- | --- | --- |
| LVFS (%) | 25.50±4.55 | 34.61± 5.88**** | 19.38± 3.63^##^ | 25.12±2.86^&&&&^ |
| LVEF (%) | 50.82±7.17 | 64.75±7.67** | 40.66± 6.38^#^ | 50.31±5.20^&&^ |
| BW(g) | 22.12±1.37 | 22.37±1.59 ^ns-1^ | 21.61±1.22 ^ns-2^ | 21.89±2.17 ^ns-3^ |
| HW (mg) | 103±9 | 122±4* | 100±7 ^ns-2^ | 106±14^&^ |
| HW/BW (mg/g) | 4.64±0.17 | 5.44±0.24** | 4.64±0.55 ^ns-2^ | 4.82±0.28^&^ |

ns-1, represents p >0.05 vs Doxorubicin+WT+ AAV9-LacZ group, *, p <0.05, **, p <0.01; ns-2, represents p >0.05 vs Doxorubicin+WT+ AAV9-LacZ group, ###, p <0.001, ####, p <0.0001; ns-3, represents p >0.05 vs Doxorubicin +FGF13-CKO+ AAV9-LacZ group, ^&^, p <0.05, ^&&^, p <0.01; LVEF, LV ejection fraction; LVFS, LV fractional shortening; HW, Height Weight; HW/BW, the ratio of height weight to body weight. All measurements are means ± SD. Data were analyzed by ANOVA with Tukey's multiple comparisons test. n.s. = not significant.

**Table S10**

Echocardiographic parameters in mouse with treatment of AAV9 (Empty Vector, and Cav1)

|  | WT+  AAV9-LacZ | WT+  AAV9 -Cav1 |
| --- | --- | --- |
| LVFS (%) | 35.35± 5.88 | 36.67±5.99 ^ns^ |
| LVEF (%) | 65.75±8.51 | 66.99±7.41 ^ns^ |
| BW(g) | 28.56±1.32 | 29.35±1.35 ^ns^ |
| HW (mg) | 136±10 | 141±11 ^ns^ |
| HW/BW (mg/g) | 4.76±0.23 | 4.80±0.16 ^ns^ |

ns, represents p >0.05 vs WT + AAV9-LacZ , *, p <0.05, LVEF, LV ejection fraction; LVFS, LV fractional shortening; HW, Height Weight; HW/BW, the ratio of height weight to body weight. All measurements are means ± SD. Data were analyzed by two-tailed unpaired Student’s t test. n.s. = not significant.

**Supplementary references**

[1] A. Heinen, S. Gödecke, U. Flögel, D. Miklos, K. Bottermann, A. Spychala, A. Gödecke . *Basic Res Cardiol*, **2021**, 116, 8.

[2] H. Xi, X. Chen, K. Liang, X. Wang, F. Jiang, Y. Li, D. Niu. *Int J Mol Sci*, **2024**, 25, 12643.

[3] Y. Ye, Z. Kuai, D. Qian, Y. He, J. Shen, K. Wu, W. Ren, Y. Hu. *Arch Gerontol Geriatr*, **2024**, 124, 105462.

[4] H. Zhao, Y. Ma, X. Zhang, H. Guo, B. Yang, R. Chi, N. Zhang, J. Wang, B. Li, F. Qin, L. Yang. *Eur J Pharmacol*, **2024**, 967, 176351.

[5] N. P. Syamprasad, S. Jain, B. Rajdev, S. R. Panda, J. K. Gangasani, V. S. Challa, J. R. Vaidya, G. C. Kundu, V. G. M. *Naidu. Chem Biol Interact*, **2023**, 381, 110566.

[6] A. C. Vandergriff, M. T. Hensley, K. Cheng. *J Vis Exp*, **2015**, 9, 52726.

[7] Y. Kuwabara, T. Horie, O. Baba, S. Watanabe, M. Nishiga, S. Usami, M. Izuhara, T. Nakao, T. Nishino, K. Otsu, T. Kita, T. Kimura, K. Ono. *Circ Res*, **2015**, 116, 279.
